# Exploring the Genetic Basis of *Calonectria* spp. Resistance in Eucalypts

**DOI:** 10.3390/cimb46100645

**Published:** 2024-09-27

**Authors:** Zhiyi Su, Wanhong Lu, Yan Lin, Jianzhong Luo, Guo Liu, Anying Huang

**Affiliations:** 1 Institute of Fast-Growing Trees, Chinese Academy of Forestry, Zhanjiang 524022, China; zhiyisu_caf_ac_cn@163.com (Z.S.); liny10000@126.com (Y.L.); luojzec@caf.ac.cn (J.L.); cercliug@caf.ac.cn (G.L.); huanganying@caf.ac.cn (A.H.); 2 College of Forestry and Grassland, Nanjing Forestry University, Nanjing 210037, China

**Keywords:** eucalypt leaf blight, resistance, allele specific expression, genetic basis

## Abstract

Selecting high-quality varieties with disease resistance by artificial crossbreeding is the most fundamental way to address the damage caused by *Calonectria* spp. in eucalypt plantations. However, understanding the mechanism of disease-resistant heterosis occurrence in eucalypts is crucial for successful crossbreeding. Two eucalypt hybrids, the susceptible EC333 (H1522 × unknown) and the resistant EC338 (W1767 × P9060), were screened through infection with *Calonectria* isolates, a pathogen that causes eucalypt leaf blight. RNA-Seq was performed on the susceptible hybrid, the disease-resistant hybrid, and their parents. The gene differential expression analysis showed that there were 3912 differentially expressed genes between EC333 and EC338, with 1631 up-regulated and 2281 down-regulated genes. The expression trends of the differential gene sets in P9060 and EC338 were similar. However, the expression trend of W1767 was opposite that of EC338. The similarity of the expression and the advantage of stress resistance in *E. pellita* suggested that genes with significant differences in expression likely relate to disease resistance. A GSEA based on GO annotations revealed that the carbohydrate binding pathway genes were differentially expressed between EC338 and EC333. The gene pathways that were differentially expressed between EC338 and EC333 revealed by the GSEA based on KEGG annotations were the sesquiterpenoid and triterpenoid biosynthesis pathways. The alternative splicing analysis demonstrated that an AS event between EC338 and EC333 occurred in LOC104426602. According to our SNP analysis, EC338 had 626 more high-impact mutation loci than the male parent P9060 and 396 more than the female parent W1767; W1767 had 259 more mutation loci in the downstream region than EC338, while P9060 had 3107 fewer mutation loci in the downstream region than EC338. Additionally, EC338 had 9631 more mutation loci in the exon region than EC333. Modules were found via WGCNA that were strongly and oppositely correlated with EC338 and EC333, such as module MEsaddlebrown, likely associated with leaf blight resistance. The present study provides a detailed explanation of the genetic basis of eucalypt leaf blight resistance, providing the foundation for exploring genes related to this phenomenon.

## 1. Introduction

### 1.1. Background and Significance of Research on Eucalypt Leaf Blight

Eucalypt leaf blight, a significant global epidemic, is triggered by fungi from the *Calonectria* genus (Ca.) of the *Nectriaceae* family [1]. Eucalypt (*Eucalyptus*, *Corymbia*, and *Angophora*) industrial plantations in Guangdong, Guangxi, Fujian, and Hainan are generally affected by eucalypt leaf blight [2]. Based on the model estimated by Zhu Jianhua et al. (2011), the average annual loss caused by eucalypt leaf blight in Fujian amounts to CNY 51.8 million [3]. Therefore, eucalypt leaf blight has become one of the most serious factors affecting the investment return and sustainable development of eucalypt plantations in China. The species diversity of the pathogenic bacteria in the genus *Calonectria* is rich, and 171 species have been reported [4,5]. Six pathogens pose a serious threat to eucalypt plantations in Guangdong and Fujian [6]. The pathogens responsible for eucalypt leaf blight are diverse and variable. Given the diverse and expansive artificial forest environment, as well as the tall and open crown of the trees, implementing effective biological and abiotic measures for control is an enormous challenge. The only solution is to select new resistant genotypes for specific environments and fundamentally solve eucalypt leaf blight.

### 1.2. Eucalyptus pellita

The high-temperature and humid summer season in South China coincides with the outbreak of eucalypt diseases. The superior disease resistance of hybrids of *E. pellita* ensures that their growth remains largely unaffected, accentuating their rapid growth [7]. Our multi-site testing results on a hybrid of *E. pellita* indicated that its rapid growth rate is not inferior to that of an *E. grandis* × *E. urophylla* hybrid, and its disease resistance is significantly higher than that of the *E. grandis* × *E. urophylla* hybrid in areas severely affected by diseases such as eucalypt leaf blight.

*E. pellita* has excellent disease resistance and displays rapid growth, and it prefers environments with strong sunlight, humid heat, and abundant rainfall [8,9,10]. *E. pellita* and its hybrids are some of the most ideal tree species for enriching eucalypt plantation species in southern China [11]. Moreover, *E. pellita* has good hybrid cross-compatibility and is trait-complementary with many other eucalypt species [12,13]. It is an important interspecific hybrid parent of eucalypt in South China. In 2007, we systematically evaluated the early introduction (from Australia and Indonesia) of *E. pellita* provenance family trials in China, and based on the evaluation, more than 200 high-performing families were selected, and an advanced-generation breeding population for *E. pellita* was established [14,15].

### 1.3. Variety Improvement Is the Solution to the Impacts of Disease

Selecting parents with different genetic bases for artificial hybridization and utilizing heterosis is an important solution for improving plant growth, biomass, stress resistance, and adaptability [16]. According to VELASCO et al.’s study, when stress was applied to the conditioned medium, heterosis was greater. The crosses had larger values than the pure strains for the resistance index [17]. The study by Maphumulo et al. showed that hybrids also displayed significantly higher levels of heterosis for both Al and MSV resistance, indicating that hybridization would be effective in generating improved varieties [18]. Strong heterosis in B. oleracea was displayed in their yield, quality, disease resistance, and stress tolerance [19]. However, the mechanism of heterosis occurrence in target traits of *E. pellita*, especially disease resistance heterosis, has not been studied yet. Therefore, exploring the molecular mechanisms of genetic recombination and gene expression patterns related to the heterosis of *E. pellita* resistant to eucalypt leaf blight and exploring the gene information related to eucalypt disease resistance would improve the development efficiency of new disease-resistant genotypes. This would be the most effective way to solve the problems of poor stress resistance (such as eucalypt leaf blight and typhoon resistance) and limited varieties used in eucalypt plantations.

The analysis of transcription levels plays a crucial role in comprehending gene expression patterns, gene architecture, and function. Transcriptome sequencing (RNA sequencing, RNA-Seq) enables the accurate acquisition of comprehensive transcriptional information and has high reproducibility [20]. Feng et al. performed a comparative transcriptome analysis between hybrids and their parents using the Illumina RNA-Seq method, proposing explanations for heterosis in sugarcane hybrids [21]. Zhao et al.’s study revealed the transcriptomic divergence of the maize F-1 hybrid and its parental lines under control and heat stress conditions and provided insight into the underlying molecular mechanisms of heterosis and the response to heat stress in maize [22]. Furthermore, RNA-Seq allows for the detection of subtle transcriptional level differences, thus facilitating a comprehensive and in-depth analysis of differentially expressed genes and their expression patterns between hybrids and parents. In Fan et al.’s study, RNA-Seq was used to identify genes related to immunity and antioxidation, providing new insights into potential strategies targeting growth traits, stress resistance, and selective breeding [23]. In Howlader et al.’s study, two different hybrids of Easter lily, obtained from two cross combinations, along with their four parents were sequenced by high-throughput RNA sequencing (RNA-Seq), finding disease resistance-conferring RPP13/1 and vicilin-like antimicrobial peptide 2-2 proteins, which were perhaps associated with plant height heterosis [24]. In the current study, two eucalypt hybrids, susceptible and resistant, were screened by leaf blight pathogen testing, which was conducted in our previous study. Transcriptome sequencing for the two hybrids and their parents was conducted using RNA-Seq so as to explore the genetic basis of the heterosis of eucalypt leaf blight resistance through gene differential expression analysis and to explore the relevant genes conferring this trait. Finally, we provide a theoretical basis for the hybrid breeding of leaf blight-resistant eucalypt.

## 2. Materials and Methods

### 2.1. Genetic Material

Eucalypt hybrid leaf blight susceptibility testing was conducted via spray inoculation with a spore suspension [5]. After 72 h of inoculation and infection, the susceptibility index (DI) of leaf blight caused by the fungus of the genus *Calonectria* on the leaves of hybrid eucalyptus plants was calculated. The percentage of the susceptible area of the tested leaves was scanned using the software Leaf Doctor [5], and the degree of susceptibility was assigned a value from 0 to 5 based on the percentage. The susceptibility index of the tested eucalypt hybrids was calculated after the statistical analyses were completed [25]. This part was completed in our previous study.

Based on our previous eucalypt leaf blight susceptibility test results [26], the eucalypt leaf blight disease-susceptible hybrid EC333 and the disease-resistant hybrid EC338 were screened, and their corresponding parents were found, for a total of 5 eucalypt genotypes used for transcriptome analysis (Table 1). There were three biological replicates for each genotype. During the peak growing season of eucalypts, healthy young leaves with consistent physiological development at the top of the trunk were collected and quickly placed in liquid nitrogen.

### 2.2. Transcriptome Sequencing and Analysis

RNA extraction: RNA was isolated utilizing the TRIzol kit (Tiangen, Beijing), and mRNA purification was carried out through oligo-T-linked magnetic beads [27]. The assessment of RNA integrity was conducted with precision employing the Agilent 2100 bioanalyzer, utilizing RNA integrity and total quantity as key reference parameters [28].

Library building and sequencing: First-strand cDNA was synthesized using a random hexamer primer and M-MuLV Reverse Transcriptase (RNase H-). Second-strand cDNA synthesis was subsequently performed using DNA Polymerase I and RNase H. Remaining overhangs were converted into blunt ends by exonuclease/polymerase. After adenylation of the 3’ ends of DNA fragments, adapters with a hairpin loop structure were ligated to prepare for hybridization. In order to select cDNA fragments of, preferentially, 370~420 bp in length, the library fragments were purified using the AMPure XP system [29]. Then, PCR was performed with Phusion High-Fidelity DNA polymerase, Universal PCR primers, and Index (X) Primer. Lastly, the PCR products were purified (AMPure XP system), and library quality was assessed using the Agilent Bioanalyzer 2100 system. The clustering of the index-coded samples was performed on a cBot Cluster Generation System using TruSeq PE Cluster Kit v3-cBot-HS (Illumia) according to the manufacturer’s instructions. After cluster generation, the prepared libraries were sequenced on an Illumina Novaseq platform, and 150 bp paired-end reads were generated [30].

Gene Differential Expression Analysis: Raw data (raw reads) in fastq format were firstly processed through fastp v0.23.0. In this step, clean data (clean reads) were obtained by removing reads containing the adapters, reads containing ploy (N), and low-quality reads from raw data. At the same time, Q20, Q30, and GC content of the clean data were calculated. All the downstream analyses were based on the high-quality clean data. Reference genome and gene model annotation files were downloaded from the genome website directly. An index of the reference genome was built using Hisat2 v2.0.5, and paired-end clean reads were aligned to the reference genome using Hisat2 v2.0.5 [31]. The mapped reads of each sample were assembled by StringTie (v1.3.3b) [32] in a reference-based approach. FeatureCounts v1.5.0-p3 was used to count the read numbers mapped to each gene. Then, the FPKM of each gene was calculated based on the length of the gene and read count mapped to this gene [33].

Differential expression analysis of two conditions was performed using the DESeq2 R package (1.20.0) (for DESeq2 with biological replicates) and the edgeR R package (3.22.5) (for edgeR without biological replicates). DESeq2 provides statistical protocols for determining differential expression in digital gene expression data using a model based on the negative binomial distribution. Unlike with DESeq2, the read counts were adjusted using the edgeR program package through one scaling-normalized factor for each sequenced library. The absolute folding change 2 was used as the threshold for significant differential expression (Benjamini and Hochberg, *p* <= 0.05) [34].

The correlation of gene expression levels among different eucalypt genotypes is a significant indicator in assessing experimental reliability and sample selection. A higher correlation coefficient indicates greater similarity and closeness in expression patterns. The ENCODE Project suggests a Pearson’s correlation coefficient *R*^2^ > 0.92 (under optimal sampling and experimental conditions) (ENCODE Project Consortium, 2004). In addition to genotype correlation, the correlation between biological replicates during testing (*R*^2^ > 0.8) is also vital for the reliability of differential gene analysis. By calculating correlation coefficients based on FPKM values and visualizing them using a heatmap, differences between sample groups and duplications within groups can be visually displayed.

Gene Ontology (GO) enrichment analysis of differentially expressed genes was implemented by the clusterProfiler R package, in which gene length bias was corrected. GO terms with corrected *p*-values less than 0.05 were considered significantly enriched by differentially expressed genes. We used the clusterProfiler R package to test the statistical enrichment of differentially expressed genes in KEGG (http://www.genome.jp/kegg/, accessed on 31 July 2023) pathways (Table S2, Figure S1) [35]. The genes were categorized based on their GO annotations according to the subnodes of the three major GO classifications (TERM) (Figure S2).

We used the local version of the GSEA tool (http://www.broadinstitute.org/gsea/index.jsp, accessed on 31 July 2023), and the GO and KEGG datasets above were used for the GSEA independently. In this investigation, the gene list for *E. grandis* was retrieved from the database to establish a network, while rMATS (4.1.0) was utilized to scrutinize alternative splicing occurrences.

Alternative splicing is an important mechanism for regulating the expression of genes and the variable of proteins. rMATS (4.1.0) software was used to analyze the AS events. GATK (v4.1.1.0) software was used to perform SNP calling. Raw vcf files were filtered with the GATK standard filter method and other parameters (cluster:3; WindowSize:35; QD < 2.0; FS > 30.0; DP < 10).

WGCNA (weighted correlation network analysis) is a systematic biological method used to describe the gene association modes among different samples. It can be used to identify gene sets that are highly synergistically changed and identify candidate biomarkers or therapeutic targets based on the coherence of gene sets and the correlation between gene sets and phenotypes. The R package WGCNA is a set of functions used to calculate various weighted associations, which can be used for network construction, gene screening, gene cluster identification, topological feature calculation, and data simulation and visualization. One input file is sample information, that is, a matrix describing the traits of the sample. The traits used for association analysis must be numeric; if a variable is regional or categorical, it needs to be converted into a 0-1 matrix. The other input file is gene expression data. For transcriptome sequencing, FPKM can be used as gene expression data [36].

## 3. Results

### 3.1. Quality Control of Sequence Data (QC)

#### 3.1.1. Sequencing Data Quality

After performing the initial data filtering (Figure S3), examining the sequencing error rates, and analyzing the GC content distribution, high-quality clean reads were obtained for further analysis. From the correspondence between base identification and the Phred scores, the evaluation of the base calling accuracy in the eucalypt samples revealed a Q20 range of 97–98% and a Q30 range of 93–94% (Table S1), indicating reliable data quality with a correct base identification rate exceeding 90%. The sequencing error rate was found to be low, ranging from 0.02 to 0.03, and the GC content distribution was stable around 50% for all the samples (Table S1). The results affirm the dependability of the sequencing data and the appropriateness of the refined reads for subsequent analysis.

#### 3.1.2. Mapping Sequencing Information to *E. grandis* Reference Genome and Its Regional Distribution

The sequencing fragments were randomly interrupted by mRNA. To determine which genes transcribed these fragments, it is necessary to compare the clean reads after quality control to the reference genome of *E. grandis* [37], a species similar to that studied in this research. A comparison was conducted between the transcriptomic data sequenced and the reference genome of Eucalyptus grandis, and the differential gene expression and functional annotations between the hybrids and parents were identified. The transcript sequencing FPKM is calculated using paired-end sequencing. A gene is represented by a pair of reads. Subsequent quantitative data analysis was performed using the number and percentage of reads aligned to the unique map of the *E. grandis* reference genome. There were 37,971,737 unique map reads for EC338. There were 34,174,054 unique map reads for EC333. There were 34,581,445 and 37,443,452 clean reads for P9060 and W1767, respectively. There were 36,358,616 clean reads for H1522 (Table S2).

The proportions of reads in the exonic regions, intronic regions, and intergenic regions of the genome were counted separately. Generally, model species with better gene annotations (e.g., humans and mice) have a high proportion of mapping to exonic regions. The distribution of the sequencing reads across genomic regions for all the eucalypt genotypes in this study showed that not many reads were mapped to intergenic regions (Figure S4), which might be due to contamination by ncRNAs or a few DNA fragments, or the gene annotations might not be well developed. The reads mapping to the reference genome might have originated from precursor mRNAs or introns stranded by variable splicing events.

### 3.2. Quantitative Analysis of the Genes Sequenced

The number of reads covered by each newly predicted gene from the start to the end region were counted based on the positional information of the genes mapped to the *E. grandis* reference genome. The reads with a mapping quality value less than 10, reads on non-composite mapping, and reads mapped to multiple regions of the genome were filtered out. The quantification of the gene expression level was performed for each sample separately and then combined to obtain the expression matrix of all the samples [38]. This enabled us to obtain the information on the genes detected in the eucalypt parents and their hybrids (Table S3).

#### 3.2.1. Detection of Expression Distribution of Genes Sequenced in Different Eucalypt Genotypes

**Figure 1:**
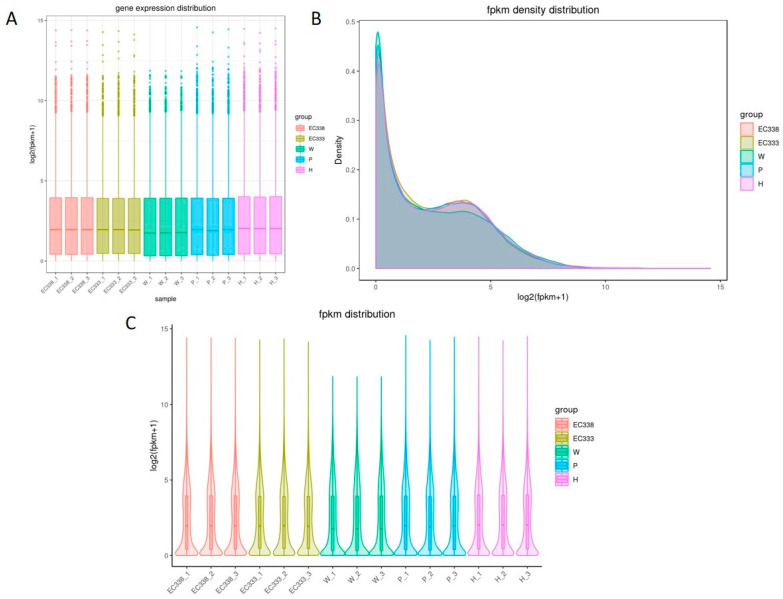
Testing the distribution of gene expression levels in eucalypt genotypes. (**A**) A boxplot of gene expression levels, where the horizontal coordinate is the name of the sample (group), and the vertical coordinate is log2(FPKM+1). (**B**) A density plot of gene expression levels, where the horizontal coordinate is log2(FPKM+1), and the vertical coordinate is the density of gene. (**C**) A violin plot of gene expression levels, where the horizontal coordinate is the name of the sample (group), and the vertical coordinate is log2(FPKM+1). The width of each violin represents the number of genes at that expression level.

Gene expression quantification using FPKM (expected number of fragments per kilobase of transcript sequence per million base pairs sequenced) is the number of fragments per million fragments per kilobase length of a gene. This metric takes into account the effects of the sequencing depth and gene length on the fragment count and converts the read count number into FPKM. It plots the overall expression distribution of the samples to view the expression level of the samples and compare the overall expression distribution of the different samples. Box plots can be utilized to illustrate the dispersion of a set of data (Figure 1A). Violin plots demonstrate the expression distribution and its probability density, with the inflated portion of the plot indicating the most concentrated region of expression in the entire sample (Figure 1C).

The results of three repeated biological tests indicated that the gene expression level distribution among the different genotypes was similar. With the exception of a slight difference in the parental line W1767, the median and the degree of dispersion of the gene expression level distribution of the four genotypes of hybrids EC338, EC333, P9060, and H1522 were relatively similar (Figure 1A,C). Therefore, the potential for differential gene screening due to the amount of gene expression can be ruled out. The peak of the FPKM density distribution curve is commonly used to analyze the gene expression level in RNA sequencing data. The peaks of the curves represent the most common FPKM values in the dataset and can provide information about the gene expression patterns. The FPKM values were calculated by dividing the number of fragments mapped to a gene by the length of the gene and the total number of mapped reads and then normalizing this to the library size.

The density distribution curve is a graphical representation of the FPKM values. The peaks of the curves represent the highest-frequency FPKM values, indicating the most common gene expression levels in the samples. The distribution of the curves for the five genotypes was generally consistent, with most of the genes having a log10 (FPKM) between 0 and 5. The first and highest peak of the curve is in close proximity to the origin; however, the density plot in this study had two peaks, indicating that two regions with the highest concentration of gene expression occurred in the five genotypes (Figure 1B). This is also reflected in the two expanded portions of Figure 1C, and in fact, Figure 1C shows a re-examination and summary of the data shown in Figure 1A,B.

#### 3.2.2. The Correlation of Gene Expression of Eucalypt Genotypes Sequenced

The gene expression correlation heatmap analysis conducted in this study (Figure S5) revealed that the eucalypt hybrids EC333 and EC338 exhibit the highest correlation coefficient (*R*^2^ > 0.9), indicating a strong similarity in their expression patterns and a close relationship between them. The high correlations observed among the three replicate groups of each genotype enhance the reliability of the subsequent differential gene analysis.

#### 3.2.3. Principal Component Analysis of Gene Expression

**Figure 2:**
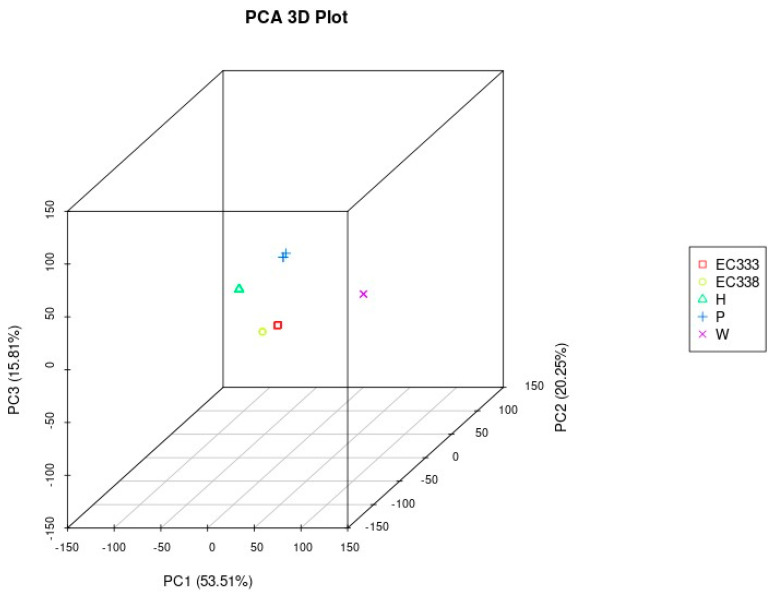
Principal component analysis plot of gene expression for each eucalypt genotype (3D).

In this study, a PCA was carried out on the gene expression values (FPKM) of all the sequenced eucalypt genotypes (Figure 2). The expected outcome of the PCA is to have samples from different groups dispersed and samples within groups clustered together in the plot. The PCA plot in Figure 2 demonstrates that the five eucalypt genotypes formed distinct clusters, reflecting their genetic relatedness. The eucalypt hybrids are clustered closely together, with a smaller distance between them compared to the distance from the parental genotypes.

### 3.3. Gene Co-Expression Venn Diagram and Differential Gene Venn Diagram

The disease-resistant eucalypt hybrid is EC338, and the susceptible hybrid is EC333. The paternal parent of EC338 is P9060 (*E. pellita*), which has good stress resistance, and the maternal parent is W1767 (*E. wetarensis*). The maternal parent of EC333 is H1522 (*E. urophylla* × *E. pellita*), and the paternal parent of EC333 is unknown, so they were not sequenced together. The disease resistance genes are most likely to be differentially expressed between the resistant genotype and susceptible genotype, and the disease resistance genes can be traced back to their parents. Therefore, in the transcriptome analysis, this study mainly compared the differential gene expression information between the resistant and susceptible genotypes, as well as between the hybrids and their parents of eucalypt hybrids, namely, EC338 vs. EC333, EC338 vs. W1767, EC338 vs. P9060, and EC333 vs. H1522.

**Figure 3:**
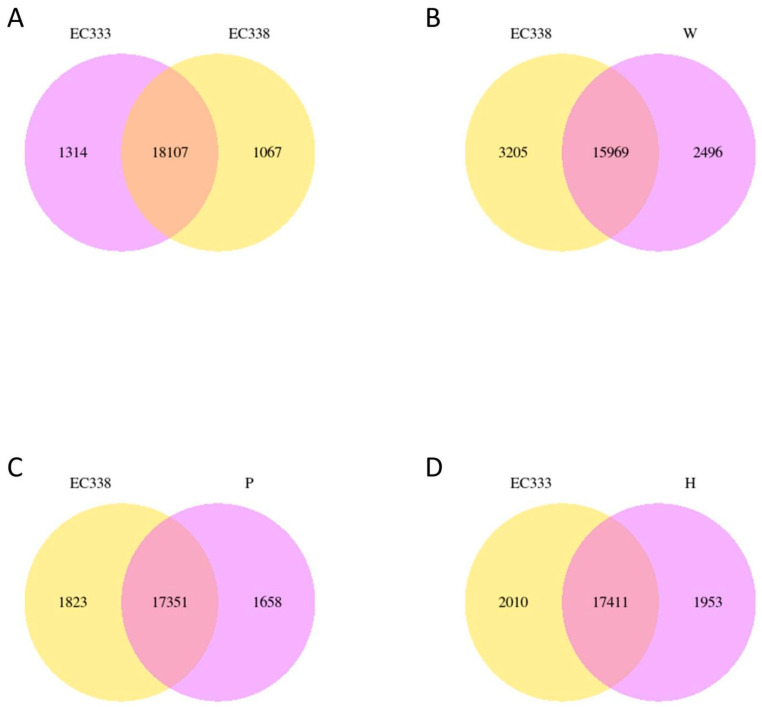
Gene co-expression Venn diagram in eucalypt genotypes tested. (Venn diagram shows the overlap of differentially expressed genes between different comparison combinations. (**A**) Venn diagram of co-expressed genes EC338 vs. EC333. (**B**) Venn diagram of co-expressed genes EC338 vs. W1767. (**C**) Venn diagram of co-expressed genes EC338 vs. P9060. (**D**) Venn diagram of co-expressed genes EC333 vs. H1522.).

**Figure 4:**
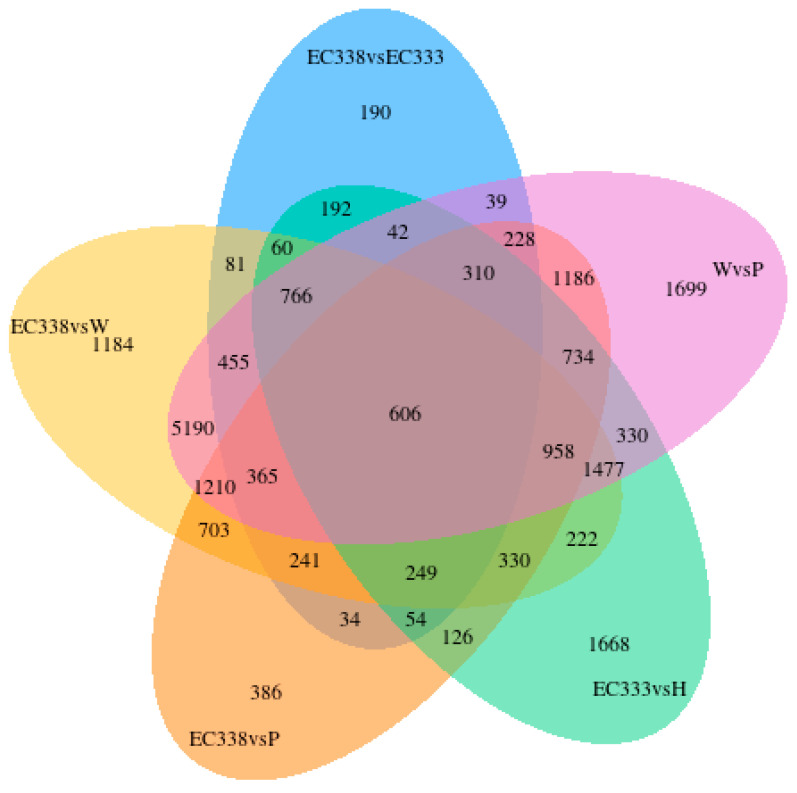
Differential gene Venn diagram in eucalypt genotypes tested.

There were 18 107 differential genes between EC338 and EC333 (Figure 3A) and 15,969 differential genes between EC338 and W1767 (Figure 3B). A total of 17,351 differential genes were identified between EC338 and P9060 (Figure 3C). Therefore, the target resistance genes might be shared by these three gene sets. In addition, genes can interact additively or reciprocally, causing the function of recessive disease susceptibility genes in a parent to become visible in their hybrids. The female parent (H1522) of EC333 is not susceptible to leaf blight, while the male parent is unknown, so its susceptibility might be attributed to gene recombination in the cross. It is unclear whether the 17,411 differential genes between EC333 and H1522 (Figure 3D) were associated with susceptibility or not. The differential gene Venn diagram clearly compares the differential genes of the multiple combinations. The sum of all the numbers in the circle represents the total number of differential genes in the comparison combination, and the overlapping area represents the common differential genes between the combinations. The four pairs of comparison groups described above (EC338 vs. EC333, EC338 vs. W1767, EC338 vs. P9060, and EC333 vs. H1522) were distinguished by 2143 common differential genes (Figure 4), indicating that these genes were likely associated with resistance to eucalypt leaf blight.

### 3.4. Gene Expression Analysis (Differential Gene Screening and Clustering)

The results indicated that 1631 genes were up-regulated, while 2281 genes were down-regulated (Figure S6). In the volcano plot, the horizontal axis represents the difference in gene expression, with the up-regulated genes depicted to the left of the dashed line as red dots and the down-regulated genes as green dots. For the samples with biological replicates, the adjusted *p*-value (padj) must be less than 0.05, which means that both the red and green dots in the volcano plot must have *p*-values above 1.301. Conversely, the smaller the corrected *p*-value is, the larger the corresponding -log10 (*p*-value) value is, indicating that the more significant the difference is between the genes in the comparison combination—and thus the greater the deviation from the horizontal axis upward—the more worthy this is of our attention. Among these genes that deviate upward along the horizontal axis, we need to find the common differential genes in the four pairs of comparative combinations mentioned above (Figure S7).

In this study, the FPKM values of the genes are clustered and analyzed using mainstream hierarchical clustering, and the genes or samples with similar expression patterns in the heatmap are clustered together. The color in each square reflects not the gene expression value but the value obtained after the homogenization (Z-score) of the rows of expression data (range of −3 to 3). Vertical comparison refers to the expression of different genes in the same sample. Horizontal comparison refers to the expression of the same gene in different samples.

**Figure 5:**
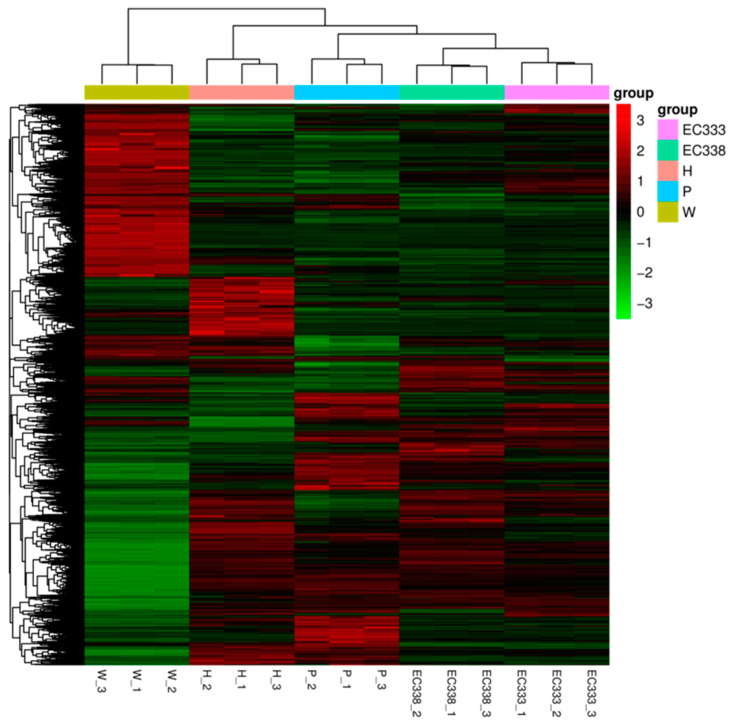
Heatmap of differentially expressed gene clustering. (Horizontal coordinates are sample names, vertical coordinates are values of differentially expressed genes after FPKM normalization. The redder the color, the higher the expression level; the greener, the lower the expression level).

The expression results among the different eucalypt genotypes revealed that numerous genes exhibited highly significant differential expression in W1767 (*E. wetarensis*). When the expression levels of the differential genes were low in W1767, they were high in EC338 (Figure 5). All of these genes with large differences in expression between the parents and hybrids were likely to be associated with disease resistance and could be the focus for the subsequent screening of resistance genes.

### 3.5. Enrichment Analysis of Differential Genes in Comparison Groups

Given the significant differences in susceptibility to eucalypt leaf blight, the primary focus of the analysis of the eucalypt leaf blight resistance genes should be on the differentially expressed genes of the resistant genotype EC338 and the susceptible genotype EC333. Furthermore, in order to investigate the inheritance pattern of the parents, the genes shared in the differential expression pathway between EC338 vs. P9060, EC338 vs. W1767, and EC333 vs. H1522 were also examined. This approach was employed in order to analyze the reasons for the development of disease resistance heterosis and attempt to identify the key genes related to eucalypt leaf blight resistance.

The GO and KEGG enrichment analysis were conducted to identify the pathways of the differentially expressed genes in the comparison group. It is important to note that only pathways with ‘details’ can be extracted to specific genes in the Gene Set Enrichment Analysis (GSEA). Therefore, we have primarily selected the pathway genes with ‘details’ after enrichment as the basis for determining the genes related to eucalypt leaf blight resistance.

**Figure 6:**
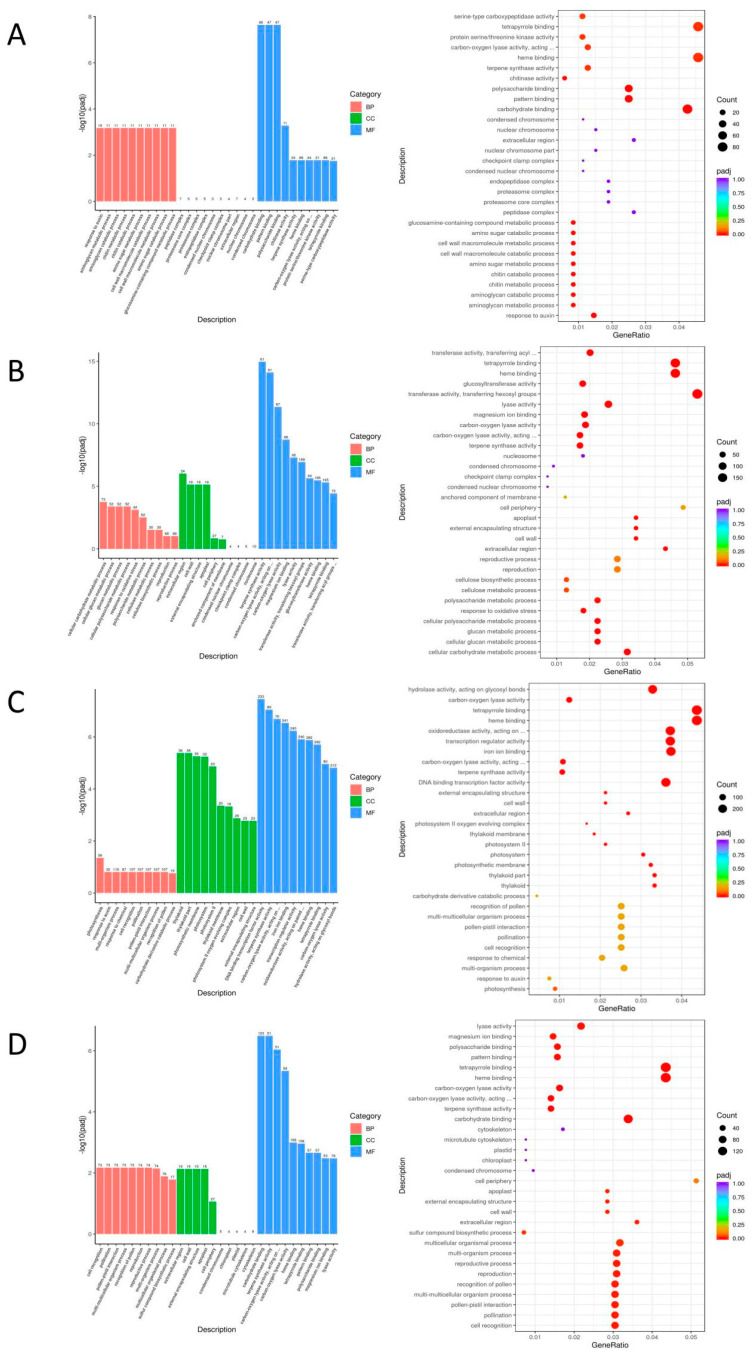
Bar (**left**) and bubble (**right**) plots of the GO analysis for four pairs of differential gene sets. (**A**) GO enrichment of EC338 vs. EC333. (**B**) GO enrichment of EC338 vs. P9060. (**C**) GO enrichment of EC338 vs. W1767. (**D**) GO enrichment of EC333 vs. H1522.

The GO enrichment analysis indicates that the differentially expressed gene pathways of EC338 (resistant) and EC333 (susceptible) predominantly involve carbohydrate binding, pattern binding, and polysaccharide binding (Table 2 and Figure 6A). These pathways might be key to endowing hybrids with disease resistance advantages, with carbohydrate binding demonstrating the highest probability, because this pathway existed in four pairs of the comparison groups, and only carbohydrate binding displayed ‘details’. Terpene synthase activity and carbon–oxygen lyase acting on phosphates were present in three pairs of genotypically differentially expressed pathways (Table 2, Figure 6B–D). The carbohydrate binding (Table 2, Figure 6A,D) and carbon–oxygen lyase activity (Table 2, Figure 6B,D) gene pathways were detected in two of the genotype pairs compared.

**Figure 7:**
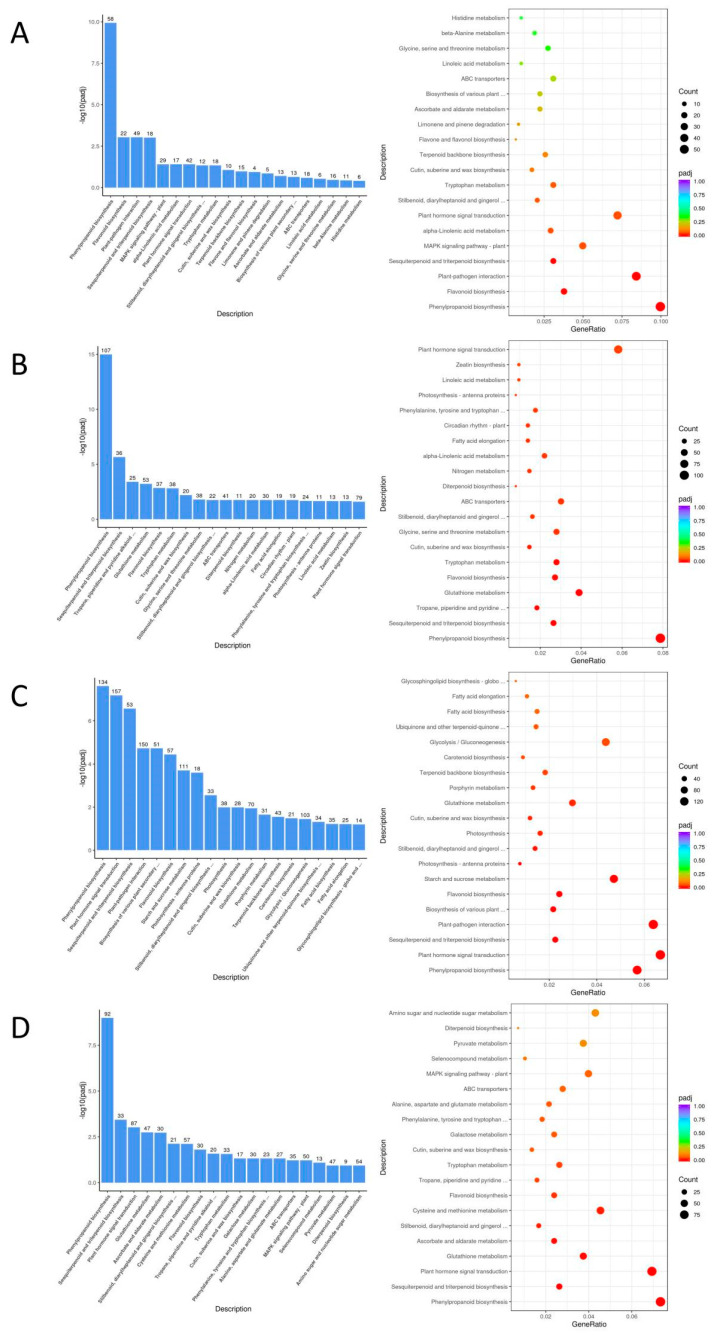
Bar (**left**) and bubble (**right**) plots of KEGG analysis for four differential gene pairs. (**A**) KEGG enrichment of EC338 vs. EC333. (**B**) KEGG enrichment of EC338 vs. P9060. (**C**) KEGG enrichment of EC338 vs. W1767. (**D**) KEGG enrichment of EC333 vs. H1522.

The KEGG enrichment analysis revealed that the differentially expressed gene pathways of EC338 (resistant) and EC333 (susceptible) included phenylpropanoid biosynthesis, flavonoid biosynthesis, plant–pathogen interaction, and sesquiterpenoid and triterpenoid biosynthesis (Table 2 and Figure 7A). These pathways were likely linked to the susceptibility to eucalypt leaf blight. Phenylpropanoid biosynthesis (EGR00940) was annotated in all four genotype pairs of differentially expressed gene pathways, while sesquiterpenoid and triterpenoid biosynthesis (EGR00909) was annotated in three pairs of genotype comparisons (Table 2, Figure 7A–C). Flavonoid biosynthesis (EGR00941) and plant–pathogen interaction (EGR04626) pathways (Table 2 and Figure 7) were annotated in two pairs of genotype comparisons (Table 2, Figure 7A,C).

**Figure 8:**
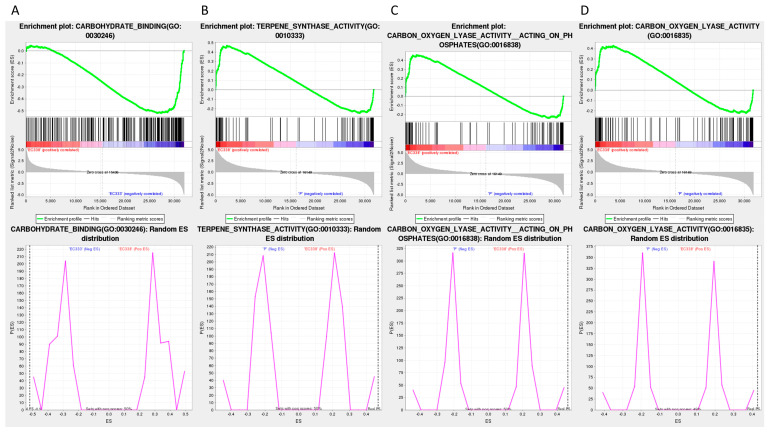
GSEA using GO annotations. (The top row is a snapshot of the enrichment results. The enrichment map displays the green ES line graph at the top, revealing the highest peak at the ES value denoting the gene set ES value. In the hits barcode map located in the middle, the leading-edge subset represents the genes that make the most significant contribution to the enrichment score, ranging from 0 to the ES value. The top grayscale enrichment of the bottom sorting level value indicates the level of genotype expression. The distribution plot in the bottom row illustrates that if the final ES value (marked by the black dotted line) is relatively small, the pathway gene set is randomly distributed, whereas a relatively large ES value suggests non-random distribution. Subsequently, the hypothesis regarding substitution is then examined. (**A**) Carbohydrate binding. (**B**) Terpene synthase activity. (**C**) Carbon–oxygen lyase acting on phosphates. (**D**) Carbon–oxygen lyase activity.).

The Gene Set Enrichment Analysis (GSEA) utilizing GO annotation primarily focused on carbohydrate binding (GO: 0030246) (Figure 8A) in EC333 in the EC338 vs. EC333 comparison, as well as terpene synthase activity (GO: 0010333) (Figure 8B), carbon–oxygen lyase acting on phosphates (GO: 0016838) (Figure 8C), and carbon–oxygen lyase activity (GO: 0016835) (Figure 8D) in EC338 in the EC338 vs. P9060 comparison. The specific differentially expressed genes that were the focus of this pathway are those listed in Tables S4–S7.

**Figure 9:**
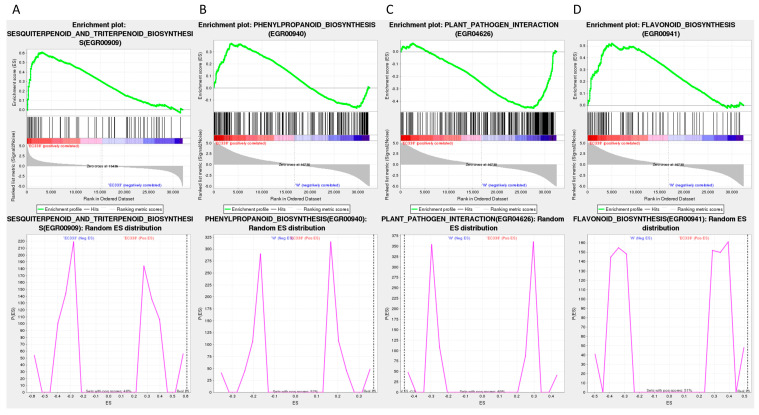
GSEA using KEGG annotations. (Here, the legend is the same as that for Figure 8. (**A**) Sesquiterpenoid and triterpenoid biosynthesis. (**B**) Phenylpropanoid biosynthesis. (**C**) Plant–pathogen interaction. (**D**) Flavonoid biosynthesis.).

The provided table illustrates that the four differentially expressed gene pathways satisfy the criteria of |NES| > 1, NOM *p*-value < 0.05, and FDR < 0.25, suggesting promising enrichment outcomes deserving further investigation (Table 3 and Table 4, Figure 8 and Figure 9).

The GSEA with KEGG annotations suggests that the main focus is on sesquiterpenoid and triterpenoid biosynthesis (EGR00909) (Figure 9A) in EC338 in the EC338 vs. EC333 comparison, plant–pathogen interaction (EGR04626) (Figure 9C) in W1767 in the EC338 vs. W1767 comparison, and phenylpropanoid biosynthesis (EGR00940) (Figure 9B) and flavonoid biosynthesis (EGR00941) (Figure 9D) in EC338 in the EC338 vs. W1767 comparison. The specific differentially expressed genes that are the focus of these pathways are listed in Tables S8–S11.

### 3.6. Alternative Splicing Event Analysis

**Figure 10:**
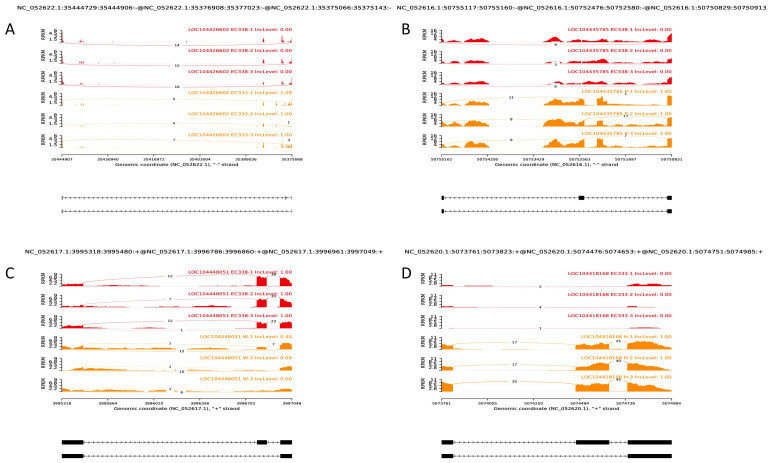
Alternative splicing SEs. (**A**) alternative splicing events of EC338 vs. EC333. (**B**) alternative splicing events of EC338 vs. P9060. (**C**) alternative splicing event of EC338 vs. W1767. (**D**) alternative splicing event of EC333 vs. H1522.

The current study identified the exon skipping events (SEs) with the most significant IncLevelDifference values in two samples from a pool of the top 20 with the lowest false discovery rate (FDR) from four comparison groups: EC338 vs. EC333, EC338 vs. P9060, EC338 vs. W1767, and EC333 vs. H1522. It was concluded that the gene LOC104426602 exhibited differential alternative splicing between EC338 and EC333 (Figure 10A), as did LOC104435785 between EC338 and P9060 (Figure 10B) and LOC104448051 between EC338 and W1767 (Figure 10C). Among the various genes with an IncLevelDifference absolute value of 1 in EC333 vs. H1522, the exon skipping isoform LOC104418168 was the only one expressed in this differential comparison group (Figure 10D). It is worth noting that LOC104426602, LOC104435785, and LOC104418168 had a high count of exon skipping isoforms, whereas LOC104448051 had a number of exon inclusion isoforms. The substantial differences in the alternative splicing events between the two distinct resistant hybrids could result in variations in the transcript and protein structures, as well as functional polymorphisms. Consequently, it was possible that the four genes mentioned are associated with resistance to eucalypt leaf blight.

### 3.7. SNP Variation Loci Analysis

**Figure 11:**
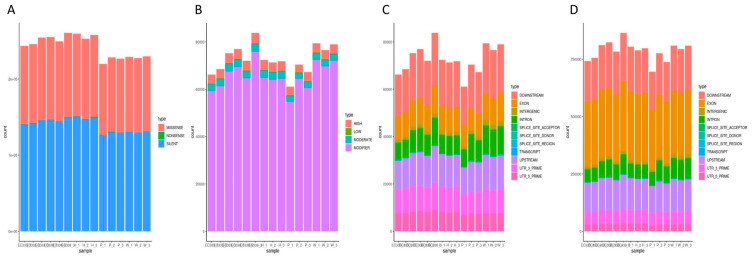
SNP variant loci region statistics. (The horizontal coordinates represent the samples. (**A**) histogram of each sample variant loci categorized based on function (SNP_function). (**B**) histogram of this by impact (INDEL_impact). (**C**) histogram of this by region (INDEL_region). (**D**) histogram of this by region (SNP_region).).

P9060 and W1767 exhibited a lower number of silent mutation loci (silent) and missense mutation loci (missense), with counts of 95917 and 98045, respectively. In contrast, the other genotypes demonstrated a higher prevalence of functional mutation loci. Missense mutations lead to the substitution of various amino acids, potentially affecting the functionality of the resultant protein. Here, we primarily focused on the 2988 distinct missense mutation loci between EC338 (resistant) and EC333 (susceptible). Notably, EC338 showed discrepancies of 12,031 and 9903 missense mutation loci compared to its parents (Figure 11A), implying a potential for the emergence of disease resistance-related genes.

INDELs usually cause more significant genetic modifications than SNPs because they involve a larger number of nucleotide sequences. INDELs were used to investigate the variant loci between the disease-resistant hybrid EC338 and its parents in the present study. It is worth noting that EC338 had 626 more impactful mutation sites than P9060 and 396 more than W1767 (Figure 11B).

The TRANSCRIPT region had the lowest number of nucleotide variations for both INDELs and SNPs. Nonetheless, scrutiny of the SNP variation loci demonstrated that the EXON region displayed the highest number of variations across all the genotypes. The INDEL variation analysis identified the DOWNSTREAM region as having a great number of variations. The analysis indicated that EC338 possessed 9631 more mutation loci in the EXON region compared to EC333. Furthermore, the female parent W1767 exhibited 259 additional mutated loci in the DOWNSTREAM region in comparison to EC338, whereas the male parent P9060 displayed 3107 fewer mutated loci in the DOWNSTREAM region than EC338. The gene loci in these regions might be related to eucalypt leaf blight and warrant further exploration.

### 3.8. Weighted Gene Co-Expression Network Analysis

**Figure 12:**
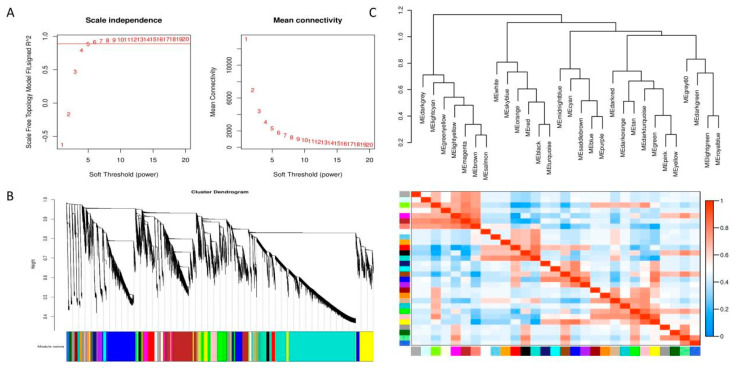
Module division in WGCNA analysis. (**A**) Soft threshold plot. In the left plot, the horizontal coordinate is the soft threshold, and the vertical coordinate is the correlation between connectivity k and p (k). The right plot is the soft threshold versus the average connectivity of the network. (**B**) Module hierarchical clustering tree. The branches are gene modules, and the leaves are genes. Merged colors are the modules with dissimilarity coefficients less than 0.25 merged with colors to represent the different modules. (**C**) Inter-module correlation heatmap. The top part of the plot is the clustering based on the module’s eigenvalues, and the values of the vertical coordinates correspond to the similarity between different modules. The upper part is the clustering based on the module eigenvalues, and the value of the vertical coordinate is the similarity between different modules. The lower part is the clustering heatmap between different modules; each row and column in the graph represents one module. The darker the color (redder) in the box, the stronger the correlation; the lighter the color of the box, the weaker the correlation.

By setting the correlation coefficient to 0.8 and the soft threshold to 5 (Figure 12A), the network was constructed in accordance with the predetermined soft threshold, and the hierarchical clustering of the genes within the network was conducted using the dissimilarity between the genes to generate a hierarchical clustering tree. Subsequently, the tree was divided into modules using dynamic shearing. The minimum number of genes in the module was 30, merging modules with correlation coefficients greater than 0.75 (the dissimilarity coefficient is less than 0.25) (Figure 12B). The 30 modules were clustered based on their eigenvalues. The extraction of the eigenvector genes of each module as the first principal component gene of a particular module also represents the overall level of gene expression within that module. Then, hierarchical clustering was performed on the characteristic genes of the different modules. The positive correlation between MEdarkgrey and MEbrown is the strongest, with a correlation coefficient of 0.55 and a *p*-value of 0.03 between the eigenvalues of the two modules. The negative correlation between MEdarkgrey and MEgrey is the strongest, with a correlation coefficient of −0.53 and a *p*-value of 0.04 for the eigenvalues of the two modules. The *p*-values of the eigenvalues for both pairs of modules were below 0.05, indicating statistical significance.

**Figure 13:**
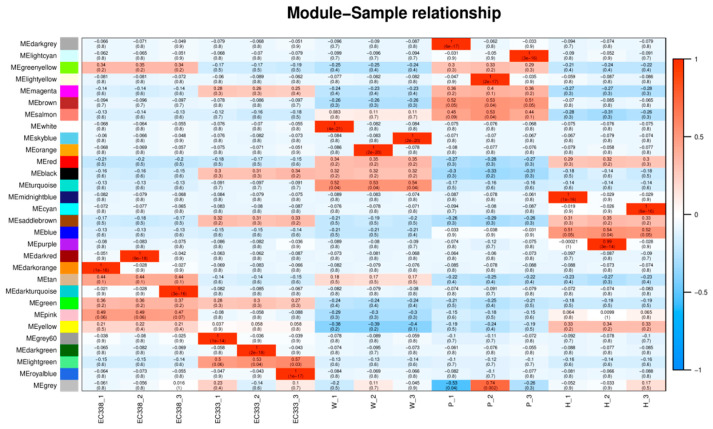
Heatmap of genotype and inter-module correlations in eucalypt genotypes. (The horizontal axis, known as the abscissa, denotes the sample, while the vertical axis, referred to as the ordinate, represents the module. Each cell in the grid contains a numerical value indicating the correlation between the module and the sample. A value closer to 1 signifies a more robust positive correlation between the module and the sample, whereas a value nearing −1 indicates a stronger negative correlation. The degree of negative correlation strength is proportional to the value. Additionally, the significance level, denoted by the value in parentheses, reflects the strength of significance, with smaller values indicating higher significance.).

The two modules with the strongest positive correlation with EC338 were MEpink and METan, with coefficients of 0.485301597 and 0.4389636, respectively. Conversely, the modules MEsaddlebrown and MEred demonstrated the most negative correlations, with coefficients of −0.204643774 and −0.17722817. When considering EC333, the modules MElightgreen and MEsaddlebrown displayed the strongest positive correlations at 0.532894171 and 0.320682081, while the modules MEred and MEgreenyellow revealed the most negative correlations at −0.177673509 and −0.170610241 (Figure 13). In theory, the same gene in the module with an opposite correlation to the two hybrid species was the gene related to eucalypt leaf blight.

It is crucial that the genes shared in the modules are positively correlated with EC338 and negatively correlated with EC333. The genes shared in the modules negatively correlated with EC338 and positively correlated with EC333 were of interest. In this regard, of these comparisons, only the MEsaddlebrown module fulfilled our criteria, so only gene 104448928 and the others in this module were of interest (Table S12).

## 4. Discussion

The screening of susceptible and resistant hybrids in this study was conducted through two infection tests on candidate eucalypt hybrids using three *Calonectria* pathogens. Based on the results of the two infection tests, the resistant genotype EC338 and the susceptible genotype EC333 were screened [26]. Although the susceptibility tests showed differences in the susceptibility index of the eucalypt hybrids to different *Calonectria* pathogens, the relative value of the susceptibility indexes of the same eucalypt hybrid after being infected twice by three pathogens was basically the same. This eliminates the randomness of the susceptibility test results, indicating that the difference in the susceptibility of the tested hybrid was due to its inherent genetic differences; that is, the disease resistance was strongly controlled by genetics. Although heterosis is generally controlled by multiple genes and the mechanisms involved in this are very complex, the genetic basis for heterosis occurrence varies among different plants, traits, and populations with different genetic backgrounds [39,40]. However, by tracking the parents of the disease-resistant hybrids and conducting combination comparisons, it is highly possible to achieve the goal of improving disease-resistant eucalypt varieties.

The expression trends of the differential gene sets in P9060 and EC338 were similar. However, the expression trend of W1767 was opposite that of EC338 (Figure 5). The similarity of the expression levels and the advantage of the stress resistance of *E. pellita* suggested that genes with significantly different expression are likely to be related to disease resistance. The expression levels of pigment genes in differently colored feather follicles were analyzed by Zheng et al., and they showed that the genetic regulation of tail feather color was independent of body plumage color [41]. The optimal genetic models were inconsistent among different lignans in the same organ and between different organs for the same lignan, which may be associated with specific gene expression patterns [42]. Abtahi et al. indicated that seed color is primarily determined by maternal genotype, but the parents’ genetic constitution determines this characteristic’s expression [43].

Studies have shown that the expression or transgenesis of carbohydrate binding modules (CBMs) could change the cell wall properties and regulate plant growth and development. For example, Keadtidumrongkul et al. found that CBM2a promoted the growth of Arabidopsis thaliana, and the growth promotion of *E. camaldulensis* was reflected in the increase in the plant height, xylem area, xylem fibers, and conduit cells, whereas there was no significant growth-promoting effect on tobacco [44]. However, there are also studies indicating that this is not always the case. For instance, the overexpression of certain CBMs did not result in the enhanced growth of *N. tabacum* [45]. Also, in the present study, the enrichment analysis based on GO annotations revealed that the differentially expressed gene pathway between the disease-resistant genotype EC338 and the susceptible genotype EC333 was associated with carbohydrate binding (GO: 0030246), indicating that CBMs might also have a correlation with leaf blight resistance in eucalypts.

In this study, a GSEA based on GO annotations revealed the differential pathway of terpene synthetase (TERPENESYNTHASEACTIVITY) in the disease-resistant hybrid EC338 compared to its parent, *E. pellita* P9060. Terpenes are critical components of plants’ defense against herbivores, insects, pathogenic fungi, and bacteria [46,47]. In many forest tree species, terpenes are vital chemical and physical defense compounds [48,49]. Conifer terpenes are a primary component of the durable, quantitative defense mechanism against stem-boring insects and fungal pathogens [50]. Constitutive and induced terpenes are critical for resistance [51,52,53]. This finding might indicate a certain correlation between terpene synthase and resistance to eucalypt leaf blight.

Furthermore, two differential pathways between EC338 and its parent P9060 were found, namely, the carbon–oxygen cleavage of phosphates (CARBON-OXYGEN-LYASE-ACTIVITY_ACTING-ON-HOSPHATES) and carbon–oxygen lyase activity (CARBON-OXYGEN-LYASE-ACTIVITY). A study showed that cold-resistant DEGs–DEPs had abundant hydrolase activities that could act on glycosyl bonds, carbon–oxygen lyase activity, and iron binding [54]. The MCRA proteins from the carbon–oxygen lyase family could be classified as FAD-containing double bond hydratases and play a role in the virulence of *Streptococcus pyogenes* M49 [55]. Based on the results of the current and other studies, it could be suggested that the above pathways are indeed related to the stress resistance mechanism of plants.

In the current study, a GSEA based on KEGG annotations revealed differential pathways in sesquiterpene and triterpenoid biosynthesis (SESQUITERPENOID-AND-TRITERPENOID-BIOSYNTHESIS) between the resistant hybrid EC338 and the susceptible hybrid EC333. Studies have shown that this pathway is related to the stress resistance mechanism of plants. For example, a study on the molecular response mechanism to drought stress in *Atractylodes chinensis* revealed 15 genes encoding enzymes involved in the biosynthesis pathways of sesquiterpenes and triterpenes [56]. Also, a finding from Singh et al. suggested a relationship between sesquiterpenes, phenolics, flavonoids, and the antioxidant capacity of *Ferula* plants [57]. Moreover, a study proposed that the synthesis of sesquiterpenes, triterpenes, flavonoids, and phenylpropanoids might play a crucial role in the response to mechanical damage in *Aquilaria sinensis* [58].

The GSEA based on KEGG annotations also revealed a differential pathway for phenylpropanoid biosynthesis (PHENYLPROPANOID_BIOSYNTHESIS) in the disease-resistant hybrid EC338 compared to its parent. Gan et al. determined that phenylpropanoid biosynthesis might play an important role in salt tolerance in mulberry at the proteomic level [59]. Additionally, a study indicated that the resistance to diseases is enhanced and disease incidence and lesion diameter is reduced in *Pichia galeiformis* through the promotion of the activation of genes involved in phenylpropanoid biosynthesis, such as *chalcone synthase* [60].

The KEGG enrichment revealed that there is a differential plant–pathogen interaction pathway (PLANT_PATHOGEN_INTERACTION) between the resistant hybrid EC338 and the susceptible hybrid EC333. Under biotic stress, plants trigger double immunity against pathogens through plant–pathogen interaction mechanisms, i.e., pathogen-associated molecular pattern-triggered immunity (PTI) and effector-triggered immunity (ETI) [61]. Recent research indicates that PTI and ETI work together to enhance plants’ immunity against pathogens, thereby enhancing their overall defense system [62,63]. Nonetheless, pathogens have developed sophisticated mechanisms to circumvent plant defenses, leading to successful infection by exploiting a host’s susceptibilities [64,65]. Whether this involves plant autoimmunity or pathogen evasion, immunity is intricately linked to the plant–pathogen interaction pathway.

In addition, the causes of plant disease resistance heterosis can also be explored through evaluating phenotypic characteristics such as the leaf surface wax, cuticle layer, morphological structure, and chlorophyll content. A recent study conducted by Bonora et al. reveals that the mechanism of resistance of *Corymbia citriodora* to different pathogens might be related to leaf phenotypic traits, such as the deposition of polyphenols and tannins on the upper/lower epidermis, and the proportion of monoterpenes, steroids, monounsaturated hydrocarbons, and long-chain hydrocarbons [66]. Also, increasing the chlorophyll content in cotton can promote photosynthesis and enhance the plant’s ability to resist diseases [67]. Therefore, in subsequent research related to this study, in addition to verifying the identified functional genes, attempts could also be made to study the different leaf phenotype characteristics between disease-resistant and -susceptible genotypes and analyze the reasons for these disease resistance differences from multiple perspectives.

The susceptibility test in the early stage of this study was conducted under controlled conditions on seedlings aged 6 months using the pathogen *Calonectria* to artificially infect and select susceptible hybrids. However, in the 1.5-year field experiment, there was no significant difference in the susceptibility between the two hybrids. This might be related to the seedlings’ age and environment. As the seedlings increase in age, the temporal expression of genes causes protein structure modification or isomerism, or different proteins might interact to resist adverse environments [68]. Yang et al. (2022) illustrated, through a comparative transcriptomic and proteomic analysis, that *P. notoginseng* mediated pathogen resistance by regulating the lignin biosynthesis pathway [69]. Therefore, the protein products most likely associated with the gene pathway associated with eucalypt leaf blight discovered in this study can be isolated, and substrate specificity and kinetic parameter analysis could be performed to further explore the leaf blight resistance mechanism.

## 5. Conclusions

In the present study, RNA sequencing was performed on disease-susceptible hybrid EC333 and disease-resistant hybrid EC338, as well as their parents, which were screened through infection with *Calonectria* (pathogenic bacteria) isolates. We selected four comparative combinations of EC338 vs. EC333, EC338 vs. W1767, EC338 vs. P9060, and EC333 vs. H1522 for a differential gene enrichment analysis. Through a Gene Set Enrichment Analysis (GSEA), we found carbohydrate binding (GO: 0030246), terpene synthase activity (GO: 0010333), carbon–oxygen lyase acting on phosphates (GO: 0016838), carbon–oxygen lyase activity (GO: 0016835), phenylpropanoid biosynthesis, flavonoid biosynthesis, plant–pathogen interaction, and sesquiterpenoid and triterpenoid biosynthesis to be differential gene pathways (Figure 8 and Figure 9) which are related to leaf blight resistance. The specific names of genes involved in these pathways have been included in Supplementary Tables (Tables S4–S11). According to an alternative splicing event analysis, the four genes LOC104426602, LOC104435785, LOC104418168, and LOC104448051 (Figure 10) were the most associated with leaf blight resistance. In light of the SNP variation loci analysis, the TRANSCRIPT region and the EXON region (Figure 11) might be related to eucalypt leaf blight resistance. The results of the weighted gene co-expression network analysis indicate that only MEsaddlebrown and gene 104448928 (Figure 12) may be associated with eucalypt leaf blight resistance.

## Figures and Tables

**Table 1:** Eucalypt leaf blight-susceptible and -resistant genotypes and their parents.

Hybrids	Susceptibility	Female	Species	Male	Species
EC338	Resistant	W1767	*E. wetarensis*	P9060	*E. pellita*
EC333	Susceptible	H1522	*E. urophylla* × *E. pellita*	Unknown	*E. urophylla*

**Table 2:** GO and KEGG enrichment analysis of four pairs of differential genes.

Comparisons	GO	KEGG
EC338 vs. EC333	Carbohydrate binding, pattern binding, polysaccharide binding (−log10(padj) > 6)	Phenylpropanoid biosynthesis, flavonoid biosynthesis, plant–pathogen interaction, sesquiterpenoid and triterpenoid biosynthesis (−log10(padj) > 2.5)
EC338 vs. P9060	Terpene synthase activity, carbon–oxygen lyase acting on phosphates, carbon−oxygen lyase activity (−log10(padj) > 10)	Phenylpropanoid biosynthesis, sesquiterpenoid and triterpenoid biosynthesis (−log10(padj) > 5)
EC338 vs. W1767	DNA binding transcription factor activity, terpene synthase activity, carbon–oxygen lyase acting on phosphates, iron ion binding, transcription regulator activity (−log10(padj) > 6)	Phenylpropanoid biosynthesis, plant hormone signal transduction, sesquiterpenoid and triterpenoid biosynthesis, plant–pathogen interaction, biosynthesis of various plant secondary, flavonoid biosynthesis (−log10(padj) > 4)
EC333 vs. H1522	Carbohydrate binding, terpene synthase activity, carbon–oxygen lyase acting on phosphates, carbon–oxygen lyase activity (−log10(padj) > 4)	Phenylpropanoid biosynthesis (−log10(padj) > 7.5)

**Table 3:** Significance test for differential gene analysis in GSEA enrichment using GO annotations.

Differential Genotypes	Genotypes	Name	Size	ES	NES	NOM *p*-Value	FDR *q*-Value	FWER *p*-Value	Rank at Max	Leading Edge
EC338 vs. EC333	EC333	Carbohydrate binding (GO:0030246)	286	−0.519	−1.580	0	0.171	0.75	5450	tags = 40%, list = 17%, signal = 48%
EC338 vs. P9060	EC338	Terpene synthase activity (GO:0010333)	86	0.469	1.952	0	0.201	0.188	2191	tags = 36%, list = 7%, signal = 39%
	EC338	Carbon–oxygen lyase acting on phosphates (GO:0016838)	89	0.462	1.939	0	0.149	0.188	2191	tags = 35%, list = 7%, signal = 37%
	EC338	Carbon–oxygen lyase activity (GO:0016835)	112	0.429	2.024	0	0.098	0.047	3561	tags = 35%, list = 11%, signal = 39%

**Table 4:** Significance test for differential gene analysis in GSEA using KEGG annotations.

Differential Genotypes	Genotypes	Name	Size	ES	NES	NOM *p*-Value	FDR *q*-Value	FWER *p*-Value	Rank at Max	Leading Edge
EC338 vs. EC333	EC338	Sesquiterpenoid and triterpenoid biosynthesis (EGR00909)	65	0.610	1.714	0	0.066	0.093	3071	tags = 35%, list = 10%, signal = 39%
EC338 vs. W1767	EC338	Phenylpropanoid biosynthesis (EGR00940)	224	0.369	1.900	0	0.048	0.048	3594	tags = 27%, list = 11%, signal = 30%
	W1767	Plant–pathogen interaction (EGR04626)	281	−0.455	−1.501	0	0.178	0.599	4752	tags = 37%, list = 15%, signal = 43%
	EC338	Flavonoid biosynthesis (EGR00941)	89	0.521	1.455	0	0.103	0.760	4985	tags = 42%, list = 15%, signal = 49%

## Data Availability

Data are contained within the article and Supplementary Materials.

## Supplementary Materials

The following supporting information can be downloaded at: https://www.mdpi.com/article/10.3390/cimb46100645/s1, Figure S1: Schematic of differential gene enrichment analysis; Figure S2: Statistical map of gene classification according to GO annotations for all genes annotated at level 2, png format (bitmap); Figure S3: Sample sequencing data filtering; Figure S4: Distribution of sequenced reads in genomic regions of eucalypt hybrids; Figure S5: Correlation heatmap of gene expression among various eucalypt genotypes; Figure S6: Histogram of the number of differential genes for each comparison combination; Figure S7: Volcano of differentially expressed genes for four pairs of genotypes. Table S1: Data quality of transcriptome sequencing of eucalypt hybrids and their parents; Table S2: Comparison of eucalypt hybrids and their parents with the E. grandis reference genome; Table S3: Genes detected in eucalypt parents and their offspring; Table S4: Genes involved in carbohydrate binding (GO-GSEA); Table S5: Genes involved in terpene synthase activity (GO-GSEA); Table S6: Genes involved in carbon–oxygen lyase acting on phosphates (GO-GSEA); Table S7: Genes involved in carbon–oxygen lyase activity (GO-GSEA); Table S8: Genes involved in sesquiterpenoid and triterpenoid biosynthesis (KEGG-GSEA); Table S9: Genes involved in phenylpropanoid biosynthesis (KEGG-GSEA); Table S10: Genes involved in plant–pathogen interaction (KEGG-GSEA); Table S11: Genes involved in flavonoid biosynthesis (KEGG-GSEA); Table S12: WGCNA disease resistance-related module genes.
