# Supplementary material for: Exploring the Genetic Basis of Calonectria spp. Resistance in Eucalypts
Source: Curr Issues Mol Biol. 2024 Sep 27;46(10):10854–79. doi: 10.3390/cimb46100645 (PMC11505705; doi:10.3390/cimb46100645)
Supplement: Supplementary file 1 [file cimb-46-00645-s001.zip › cimb-3189246-supplementary.pdf]

$$P = 1 - \sum_{i=0}^{m-1} \frac{\binom{M}{i}\binom{N-M}{n-i}}{\binom{N}{n}}$$

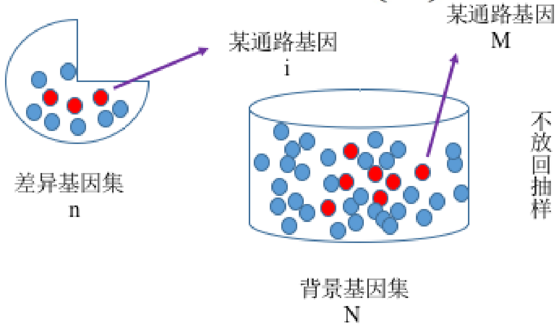

Figure S1. Schematic of differential gene enrichment analysis.

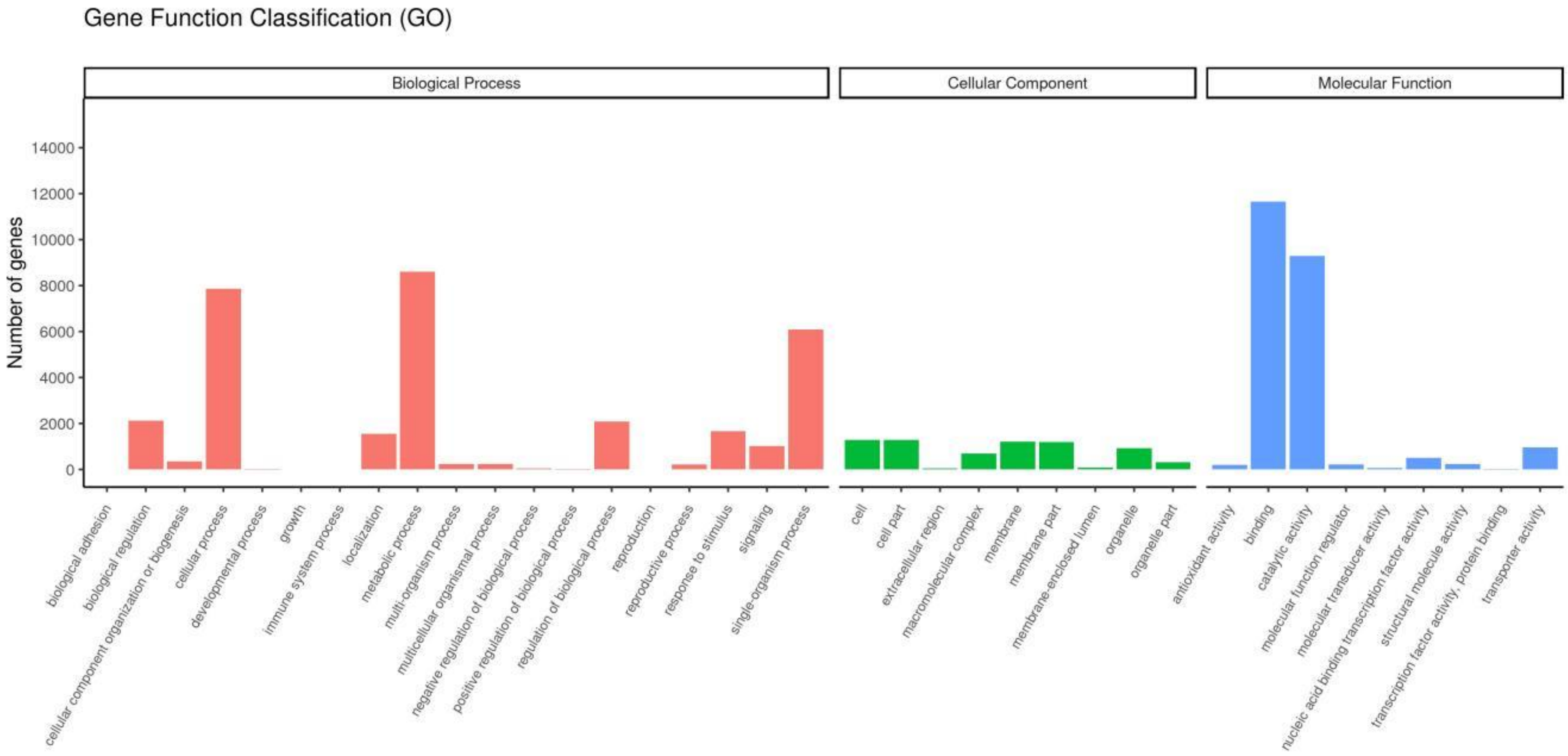

Figure S2. Statistical map of gene classification according to GO annotations for all genes annotated at level 2, png format (bitmap).

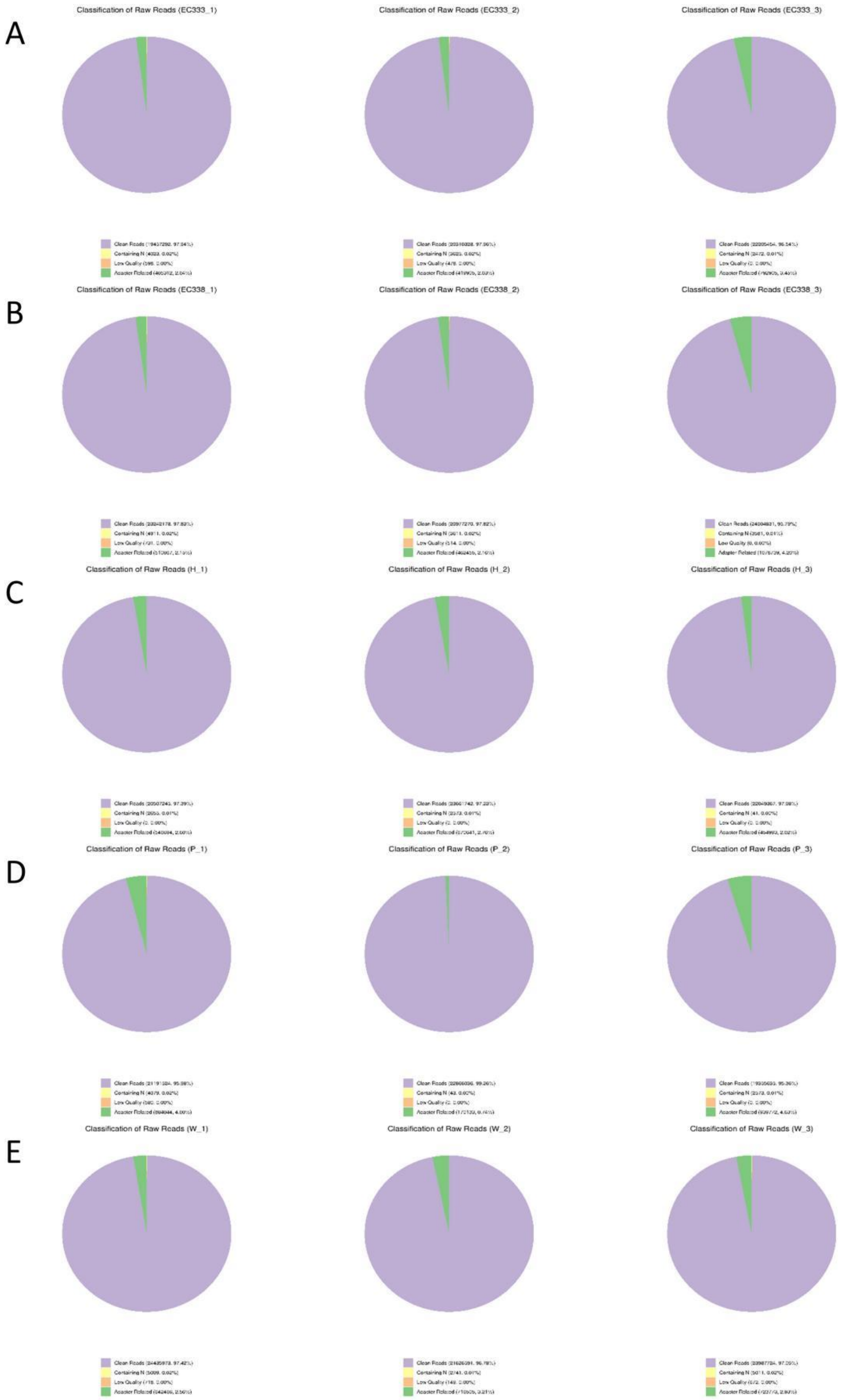

Figure S3. A-E Sample sequencing data filtering.

Adapter related: percentage of reads with adapters, Containing N: percentage of reads with N bases, Low quality: percentage of reads with low sequencing quality, Clean reads: percentage of clean reads.

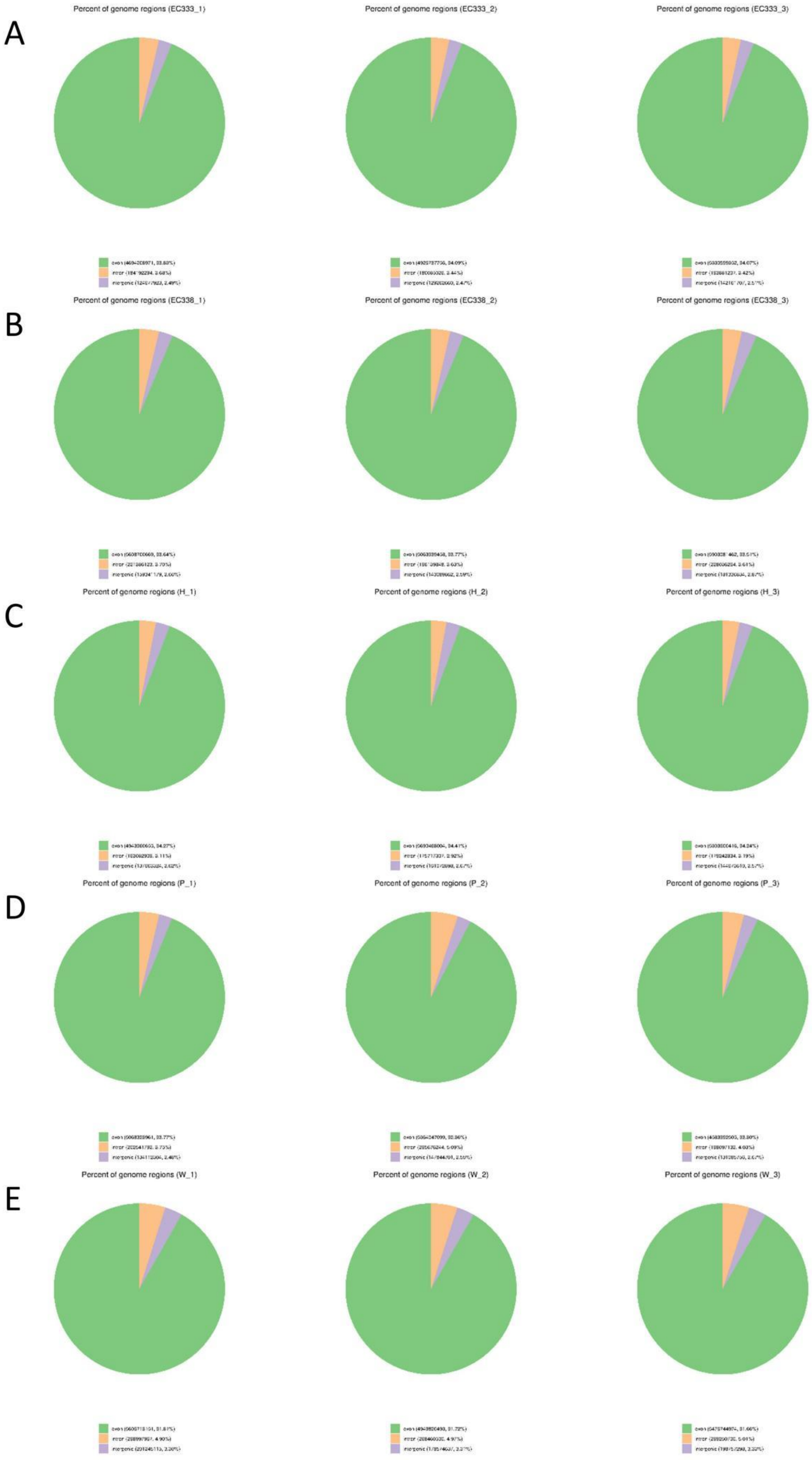
A
Percent of genome regions (EC333_1)
Percent of genome regions (EC333_2)
Percent of genome regions (EC333_3)
B
Percent of genome regions (EC338_1)
Percent of genome regions (EC338_2)
Percent of genome regions (EC338_3)
C
Percent of genome regions (H_1)
Percent of genome regions (H_2)
Percent of genome regions (H_3)
D
Percent of genome regions (P_1)
Percent of genome regions (P_2)
Percent of genome regions (P_3)
E
Percent of genome regions (W_1)
Percent of genome regions (W_2)
Percent of genome regions (W_3)

Figure S4. A-E Distribution of sequenced reads in genomic regions of eucalypt hybrids.

Exon: the number of bases compared to the exonic region of the genome and its percentage of the number of bases compared to the reference genome, Intron: the number of bases compared to the intronic region of the genome and its percentage of the number of bases compared to the reference genome, Intergenic: the number of bases compared to the intergenic region and its percentage of the number of bases compared to the reference genome. Intergenic: the number of bases matched to intergenic regions and their percentage of the number of bases matched to the reference genome.

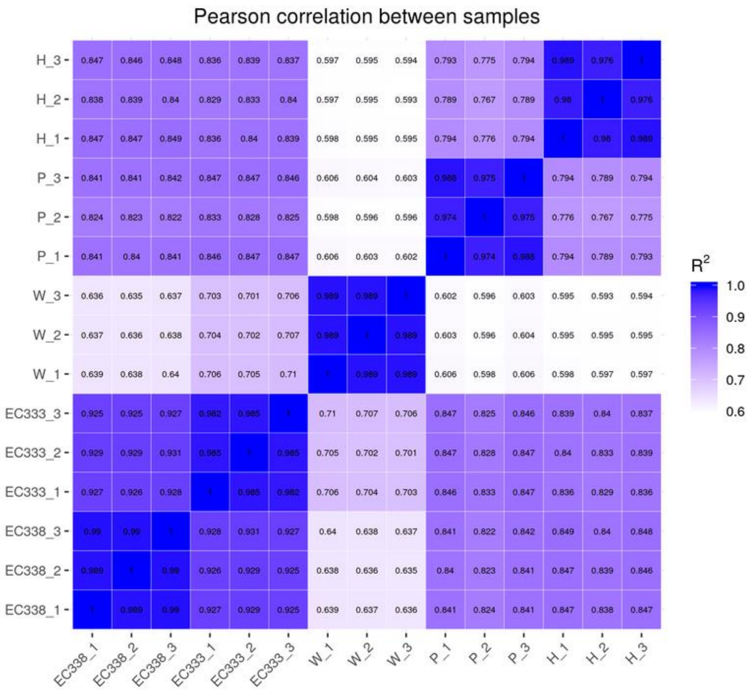

Figure S5. Correlation heat map of gene expression among various eucalypt genotypes (the horizontal and vertical coordinates are the square of the correlation coefficient between each genotype).

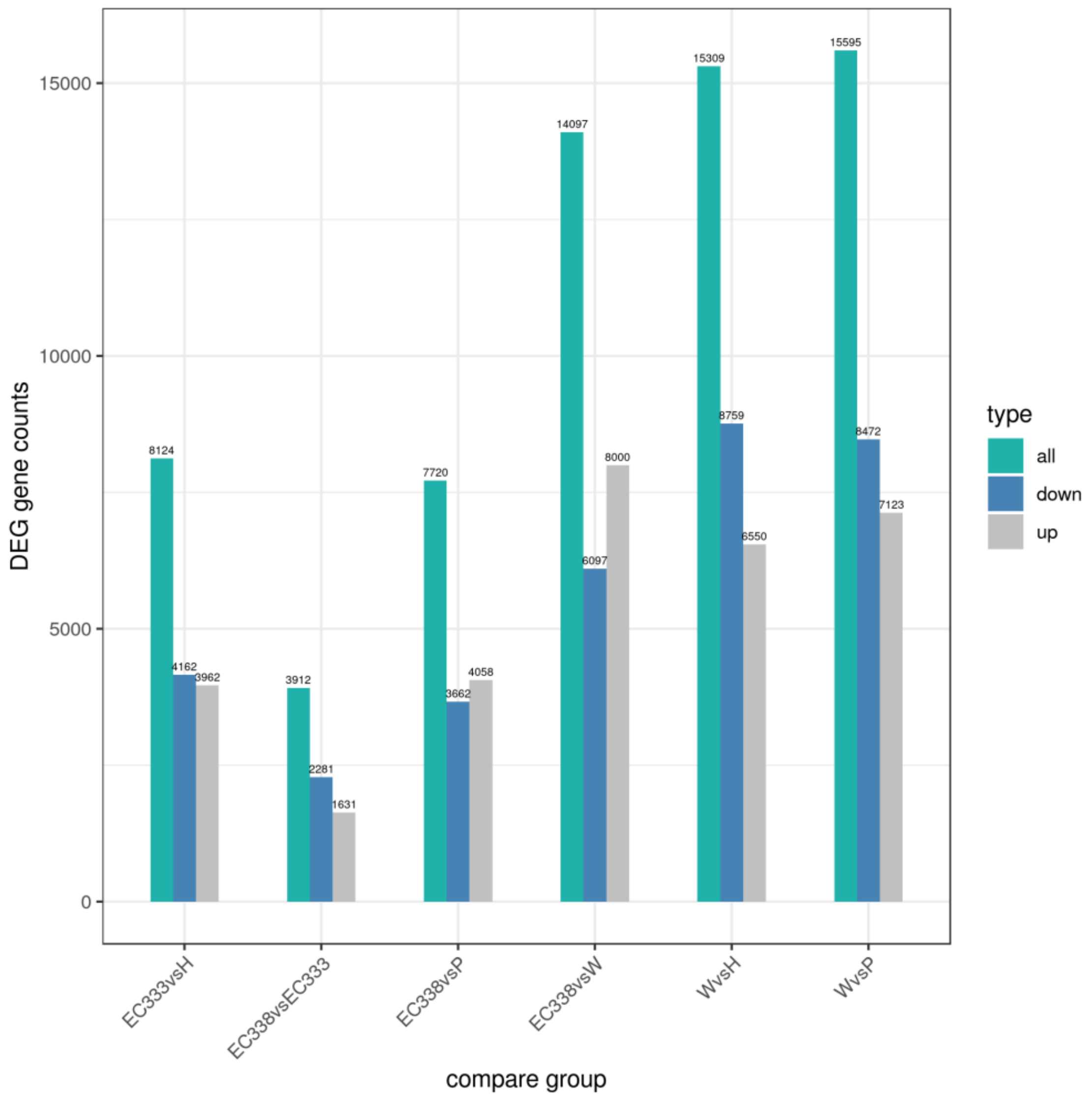

Figure S6. Histogram of the number of differential genes for each comparison combination (blue and gray indicate up- and down-regulated genes, respectively, and numbers indicate the number of genes).

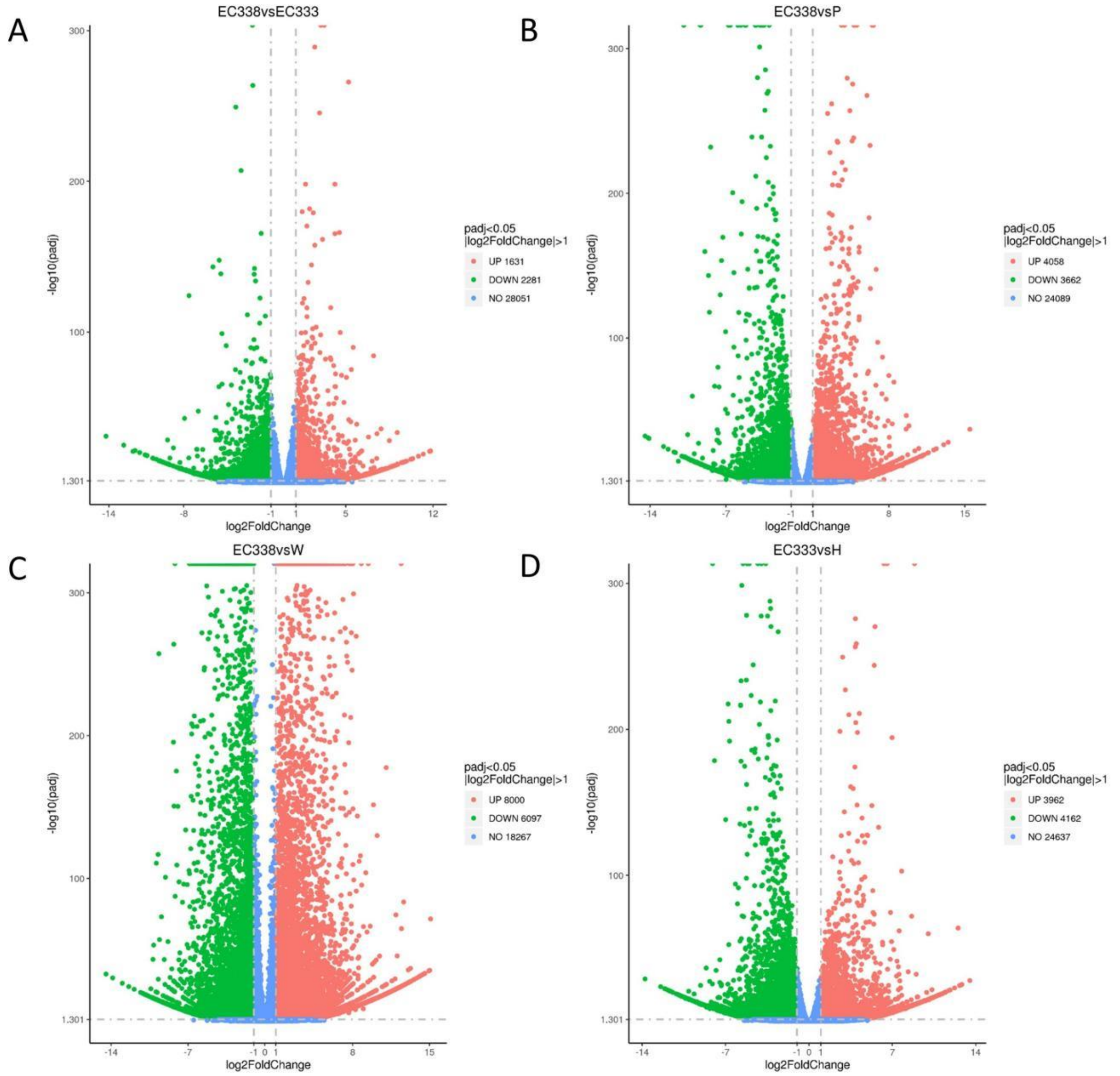

Figure S7. A-D Volcano of differentially expressed genes for four pairs of genotypes (the more off-center the horizontal axis is, the greater the multiplicity of differences; the more the vertical axis is toward the top of the plot, the more significant the differences are; the dashed line indicates the threshold line for the differential gene screening criteria).

Table S1. Data quality of transcriptome sequencing of eucalypt hybrids and their parents.

Sample	Library	Raw Reads	Raw Bases	Clean Reads	Clean Bases	Error Rate	Q20	Q30	GC pct %
EC338-1	FRAS230192178-1A	47515774	7.13G	46484356	6.97G	0.02	98.05	94.73	51.09
EC338-2	FRAS230192179	42887700	6.43G	41954540	6.29G	0.02	97.93	94.46	51.19

	-1A								
EC338-3	FRAS230209768-1A	51374302	7.71G	49209662	7.38G	0.02	98	94.38	51.19
EC333-1	FRAS230192180-1A	39734450	5.96G	38914584	5.84G	0.02	98.06	94.75	50.58
EC333-2	FRAS230192181-1A	41468672	6.22G	40620656	6.09G	0.02	97.96	94.5	51.21
EC333-3	FRAS230209769-1A	46001662	6.9G	44410908	6.66G	0.03	97.48	93.53	51.64
W-1	FRAS230192182-1A	50168332	7.53G	48871946	7.33G	0.02	98.1	94.78	49.68
W-2	FRAS230192183-1A	44691972	6.7G	43253182	6.49G	0.02	97.95	94.59	49.8
W-3	FRAS230192184-1A	49434360	7.42G	47975448	7.2G	0.02	97.96	94.42	49.66
P-1	FRAS230192185-1A	44160454	6.62G	42383048	6.36G	0.02	97.9	94.39	50.81
P-2	XRAS230006854-4r	46072424	6.91G	45732072	6.86G	0.03	97.82	93.73	48.75
P-3	FRAS230209770-1A	40595960	6.09G	38711270	5.81G	0.03	97.54	93.71	50.63
H-1	FRAS230209771-1A	42113188	6.32G	41014490	6.15G	0.03	97.61	93.81	51.65
H-2	FRAS230209812-1A	48669912	7.3G	47323484	7.1G	0.03	97.55	93.82	52.18
H-3	XRAS230006855-2r	45008802	6.75G	44098734	6.61G	0.03	97.65	93.82	51.72

Notes: Sample: Sample name. Library: Library number. Raw Reads: Number of reads in the original dataset. Raw Bases: Number of bases in the original dataset(raw base=raw reads*150bp). Clean Reads：Number of reads after filtering raw data. Clean Bases：Number of reads after filtering raw data(clean base=clean reads*150bp). Error Rate: The data's overall sequencing error rate. Q20: Percentage of total bases with Phred values greater than 20. Q30: Percentage of total bases with Phred

values greater than 30. GC pct%: The percentage of G versus C in clean reads is calculated for all four bases.

Table S2. Presents a comparison of eucalypt hybrids and their parents with the E. grandis reference genome.

Sample	Total map (n/%)	Unique map (n/%)	Multi map (n/%)	read1 map (n/%)	read2 map (n/%)	Positive map (n/%)	Negative map (n/%)	Splice map (n/%)	Unsplice map (n/%)	Proper map (n/%)
EC338-1	40159966 /86.39	38558167 /82.95	1601799/ 3.45	19237051 /41.38	19321116 /41.56	19247863 /41.41	19310304 /41.54	14669935 /31.56	23888232 /51.39	34823836 /74.92
EC338-2	36211213 /86.31	34796756 /82.94	1414457/ 3.37	17370036 /41.4	17426720 /41.54	17363332 /41.39	17433424 /41.55	13227206 /31.53	21569550 /51.41	31446686 /74.95
EC338-3	42338677 /86.04	40560287 /82.42	1778390/ 3.61	20271145 /41.19	20289142 /41.23	20242786 /41.14	20317501 /41.29	15323738 /31.14	25236549 /51.28	36530762 /74.23
EC333-1	33544701 /86.2	32214360 /82.78	1330341/ 3.42	16070400 /41.3	16143960 /41.49	16078334 /41.32	16136026 /41.47	12578129 /32.32	19636231 /50.46	29221844 /75.09
EC333-2	35104775 /86.42	33762517 /83.12	1342258/ 3.3	16855306 /41.49	16907211 /41.62	16854963 /41.49	16907554 /41.62	12976420 /31.95	20786097 /51.17	30543652 /75.19
EC333-3	38005714 /85.58	36545285 /82.29	1460429/ 3.29	18276630 /41.15	18268655 /41.14	18226461 /41.04	18318824 /41.25	13812660 /31.1	22732625 /51.19	33019178 /74.35
W-1	40966127 /83.82	39255844 /80.32	1710283/ 3.5	19577756 /40.06	19678088 /40.26	19604178 /40.11	19651666 /40.21	14318448 /29.3	24937396 /51.03	34911134 /71.43
W-2	36207525 /83.71	34720281 /80.27	1487244/ 3.44	17359942 /40.14	17360339 /40.14	17341110 /40.09	17379171 /40.18	12643301 /29.23	22076980 /51.04	30911876 /71.47
W-3	40077327 /83.54	38354230 /79.95	1723097/ 3.59	19151315 /39.92	19202915 /40.03	19153905 /39.92	19200325 /40.02	13988035 /29.16	24366195 /50.79	34094218 /71.07
P-1	36247769 /85.52	34844811 /82.21	1402958/ 3.31	17394413 /41.04	17450398 /41.17	17388937 /41.03	17455874 /41.19	13905270 /32.81	20939541 /49.41	31567568 /74.48
P-2	38936463 /85.14	37274764 /81.51	1661699/ 3.63	18811318 /41.13	18463446 /40.37	18635037 /40.75	18639727 /40.76	15579472 /34.07	21695292 /47.44	32493534 /71.05
P-3	32957503 /85.14	31624759 /81.69	1332744/ 3.44	15797846 /40.81	15826913 /40.88	15783669 /40.77	15841090 /40.92	12584014 /32.51	19040745 /49.19	28631266 /73.96
H-1	35171695 /85.75	33842523 /82.51	1329172/ 3.24	16892358 /41.19	16950165 /41.33	16891991 /41.19	16950532 /41.33	12644630 /30.83	21197893 /51.68	30650360 /74.73
H-2	40426354 /85.43	38905691 /82.21	1520663/ 3.21	19384760 /40.96	19520931 /41.25	19405029 /41.01	19500662 /41.21	14155675 /29.91	24750016 /52.3	36117940 /76.32
H-3	37735187 /85.57	36327633	1407554/ 3.19	18204108 /41.28	18123525 /41.1	18141086 /41.14	18186547 /41.24	13587218 /30.81	22740415 /51.57	32718452 /74.19

Notes: Total Reads: After quality control, the sequencing data yielded a specific number of clean reads. Total map: The number of reads matched to the genome and their percentage. Unique map: The percentage and count of reads that aligned to a unique position in the reference genome, for further

quantitative data analysis. Multi map: The quantity of sequencing reads and the corresponding proportion that underwent alignment against various positions within the genomic reference were scrutinized. read1 map: the number of read1s compared to the reference genome and their percentage, read2_map: the number of read2s compared to the reference genome and their percentage. Positive map: The quantity of reads and their respective proportion in relation to the positive strand of the reference genome is under examination. Negative_map：The quantity of sequences and their corresponding proportion when juxtaposed with the antisense strand of the foundational genetic blueprint. Splice map: The quantity and proportion of reads demonstrating a split-matched alignment with the genomic reference sequence. Unsplice map: The quantity of reads that did not align with the genome and their respective percentage. Proper map: The alignment of read pairs (read1 and read2) was conducted concurrently with an evaluation of their frequencies and proportions within the genomic sequence.

Table S3. Genes detected in eucalypt parents and their offspring.

Gene id	Gene name	Gene chr	Gene start	Gene end	Gene strand	Gene length	Gene biotype	Gene description	Sub-family	Family
104422107	LOC104422107	NC_052621.1	18302801	18304215	+	886	protein_coding	ribulose bisphosphate carboxylase small chain%2C chloroplastic && sp|P24007|RBS_PYRPY Ribulose bisphosphate carboxylase small subunit, chloroplastic OS=Pyrus pyrifolia OX=3767 GN=RBCS PE=2 SV=1 && PF12338:Ribulose-1,5-bisphosphate carboxylase small subunit|PF00101:Ribulose bisphosphate carboxylase, small chain	-	-
104444821	LOC104444821	NC_052616.1	32742793	32743761	-	969	protein_coding	chlorophyll a-b binding protein of LHCII type 1 && sp|P27493|CB22_TOBAC Chlorophyll a-b binding protein 21, chloroplastic OS=Nicotiana tabacum OX=4097 GN=CAB21	-	-

								PE=2 SV=1 && PF00504:Chlorophyll A-B binding protein		
1044 2510 7	LOC 1044 2510 7	NC_ 0526 22.1	1663 1474	1663 2275	+	636	prot ein_ codi ng	non-specific lipid-transfer protein 1 && sp｜P85894｜LTP1_MORNI Non-specific lipid-transfer protein 1 OS=Morus nigra OX=85232 PE=1 SV=1 && PF00234:Protease inhibitor/seed storage/LTP family	-	-
1044 3469 2	LOC 1044 3469 2	NC_ 0526 12.1	3456 0961	3456 2840	-	1068	prot ein_ codi ng	chlorophyll a-b binding protein 36%2C chloroplastic && sp｜P27518｜CB21_GOSHI Chlorophyll a-b binding protein 151, chloroplastic OS=Gossypium hirsutum OX=3635 GN=CAB-151 PE=2 SV=2 && PF00504:Chlorophyll A-B binding protein	-	-

Notes: Gene id: Gene count. Sample: The quantification of each sample yielded a raw READ COUNT value.Gene name: The nomenclature of the genetic entity. Gene chr：The gene is located on a specific chromosome. Gene start: The gene's location on the chromosome is determined by its starting position. Gene end: The chromosomal locus where a gene is situated terminates at a specific position on the chromosome. Gene strand: Details regarding the positive and negative strands of the chromosome harboring the gene are being discussed. Gene length: The gene length is defined as the cumulative measure of all non-overlapping exon regions spanning from the initiation to the termination of a gene. Gene biotype: Various types of genes exist, including protein-coding genes, long-chain non-coding genes, among others. Gene description: The gene function description is detailed alongside reference genome annotation information preceding the initial && symbol, Swiss-Prot database annotation following the first && symbol, and Pfam database annotation subsequent to the second && symbol. Tf family：Annotation of gene transcription factor families.

Table S4. Genes in carbohydrate binding (GO-GSEA).

	PROBE	DESCRIPTION (from dataset)	GENE SYMBOL	GENE_TITLE	RANK IN GENE LIST	RANK METRIC SCORE	RUNNING ES	CORE ENRICHMENT
172	104451739	LOC104451739			26514	-0.802	-0.5163	Yes
173	104427375	LOC104427375			26517	-0.802	-0.514	Yes
174	120296231	LOC120296231			26644	-0.819	-0.5156	Yes
175	104425265	LOC104425265			26693	-0.823	-0.5146	Yes
176	120289673	LOC120289673			26739	-0.834	-0.5136	Yes
177	120291737	LOC120291737			26849	-0.847	-0.5145	Yes
178	120296079	LOC120296079			26857	-0.847	-0.5122	Yes
179	120292471	LOC120292471			26895	-0.851	-0.5109	Yes
180	104418351	LOC104418351			26948	-0.858	-0.51	Yes
181	104442507	LOC104442507			26995	-0.859	-0.5089	Yes
182	120292993	LOC120292993			27300	-0.89	-0.5159	Yes
183	104419218	LOC104419218			27326	-0.895	-0.514	Yes
184	104428058	LOC104428058			27408	-0.913	-0.5139	Yes
185	104427280	LOC104427280			27455	-0.921	-0.5126	Yes
186	104416546	LOC104416546			27523	-0.935	-0.5119	Yes
187	120296236	LOC120296236			27527	-0.936	-0.5093	Yes
188	104437884	LOC104437884			27534	-0.937	-0.5067	Yes
189	104419425	LOC104419425			27716	-0.971	-0.5095	Yes
190	120287022	LOC120287022			27847	-0.988	-0.5107	Yes
191	120295547	LOC120295547			27876	-0.993	-0.5086	Yes
192	120289765	LOC120289765			28053	-1.033	-0.5111	Yes
193	104424490	LOC104424490			28094	-1.041	-0.5093	Yes
194	104425264	LOC104425264			28144	-1.054	-0.5077	Yes
195	104450896	LOC104450896			28145	-1.054	-0.5046	Yes
196	104427563	LOC104427563			28282	-1.087	-0.5057	Yes
197	120290470	LOC120290470			28342	-1.101	-0.5043	Yes
198	104453202	LOC104453202			28611	-1.164	-0.5093	Yes
199	104435019	LOC104435019			28626	-1.169	-0.5063	Yes
200	104442597	LOC104442597			28659	-1.177	-0.5038	Yes
201	104425585	LOC104425585			28758	-1.202	-0.5034	Yes
202	104457009	LOC104457009			28782	-1.208	-0.5005	Yes
203	104452347	LOC104452347			28799	-1.213	-0.4974	Yes
204	104455878	LOC104455878			28840	-1.226	-0.4951	Yes
205	120288503	LOC120288503			28859	-1.231	-0.492	Yes
206	104435473	LOC104435473			28964	-1.266	-0.4915	Yes
207	104420744	LOC104420744			29015	-1.284	-0.4893	Yes
208	120294558	LOC120294558			29022	-1.285	-0.4857	Yes
209	108957345	LOC108957345			29054	-1.297	-0.4828	Yes
210	104437043	LOC104437043			29392	-1.423	-0.4893	Yes
211	120291983	LOC120291983			29424	-1.436	-0.486	Yes
212	104446144	LOC104446144			29432	-1.439	-0.4819	Yes

213	120289594	LOC120289594	29510	-1.468	-0.48	Yes
214	104442589	LOC104442589	29696	-1.554	-0.4813	Yes
215	104424015	LOC104424015	29716	-1.563	-0.4773	Yes
216	104453182	LOC104453182	29777	-1.597	-0.4744	Yes
217	104414070	LOC104414070	29827	-1.622	-0.4712	Yes
218	104427371	LOC104427371	29834	-1.624	-0.4665	Yes
219	120289599	LOC120289599	29986	-1.707	-0.4663	Yes
220	120294795	LOC120294795	29999	-1.713	-0.4616	Yes
221	104416038	LOC104416038	30004	-1.716	-0.4566	Yes
222	104425283	LOC104425283	30012	-1.72	-0.4517	Yes
223	104431708	LOC104431708	30124	-1.791	-0.45	Yes
224	104431212	LOC104431212	30177	-1.823	-0.4462	Yes
225	104416068	LOC104416068	30181	-1.824	-0.4409	Yes
226	104420740	LOC104420740	30192	-1.831	-0.4358	Yes
227	104434429	LOC104434429	30202	-1.835	-0.4306	Yes
228	120296238	LOC120296238	30220	-1.844	-0.4257	Yes
229	120293387	LOC120293387	30233	-1.852	-0.4206	Yes
230	104416600	LOC104416600	30282	-1.888	-0.4165	Yes
231	104427057	LOC104427057	30306	-1.901	-0.4116	Yes
232	104452523	LOC104452523	30314	-1.907	-0.4062	Yes
233	104453353	LOC104453353	30339	-1.919	-0.4013	Yes
234	104417063	LOC104417063	30396	-1.965	-0.3973	Yes
235	104427276	LOC104427276	30462	-2.011	-0.3934	Yes
236	120294798	LOC120294798	30519	-2.059	-0.389	Yes
237	104457401	LOC104457401	30538	-2.089	-0.3834	Yes
238	104440880	LOC104440880	30558	-2.107	-0.3778	Yes
239	104455879	LOC104455879	30578	-2.127	-0.3721	Yes
240	104433328	LOC104433328	30587	-2.134	-0.366	Yes
241	104440043	LOC104440043	30590	-2.137	-0.3598	Yes
242	104451077	LOC104451077	30618	-2.158	-0.3542	Yes
243	104449039	LOC104449039	30632	-2.168	-0.3482	Yes
244	104451920	LOC104451920	30766	-2.271	-0.3457	Yes
245	120289800	LOC120289800	30869	-2.376	-0.3419	Yes
246	104452592	LOC104452592	30882	-2.395	-0.3352	Yes
247	104440029	LOC104440029	30901	-2.412	-0.3286	Yes
248	104430597	LOC104430597	30985	-2.503	-0.3238	Yes
249	104442603	LOC104442603	30994	-2.506	-0.3166	Yes
250	108960421	LOC108960421	31005	-2.511	-0.3095	Yes
251	104455436	LOC104455436	31056	-2.555	-0.3035	Yes
252	104438704	LOC104438704	31057	-2.557	-0.296	Yes
253	104440879	LOC104440879	31061	-2.563	-0.2885	Yes
254	104430169	LOC104430169	31079	-2.584	-0.2814	Yes
255	104429110	LOC104429110	31085	-2.591	-0.2739	Yes
256	104424563	LOC104424563	31098	-2.614	-0.2665	Yes
257	104451790	LOC104451790	31113	-2.631	-0.2592	Yes
258	104451714	LOC104451714	31175	-2.724	-0.253	Yes
259	104447342	LOC104447342	31190	-2.741	-0.2454	Yes
260	120291350	LOC120291350	31194	-2.753	-0.2373	Yes
261	104456007	LOC104456007	31198	-2.759	-0.2292	Yes

262	104427378	LOC104427378			31211	-2.779	-0.2214	Yes
263	104428611	LOC104428611			31229	-2.802	-0.2136	Yes
264	104446289	LOC104446289			31249	-2.835	-0.2058	Yes
265	104421788	LOC104421788			31261	-2.85	-0.1977	Yes
266	104452340	LOC104452340			31274	-2.867	-0.1896	Yes
267	104434436	LOC104434436			31285	-2.885	-0.1814	Yes
268	104427372	LOC104427372			31298	-2.902	-0.1732	Yes
269	120286921	LOC120286921			31389	-3.09	-0.1669	Yes
270	120294680	LOC120294680			31401	-3.108	-0.1581	Yes
271	104428452	LOC104428452			31405	-3.113	-0.1489	Yes
272	104427380	LOC104427380			31412	-3.12	-0.1399	Yes
273	120286632	LOC120286632			31423	-3.148	-0.1309	Yes
274	104427381	LOC104427381			31430	-3.16	-0.1217	Yes
275	104456032	LOC104456032			31462	-3.235	-0.1131	Yes
276	120286923	LOC120286923			31473	-3.259	-0.1038	Yes
277	104428449	LOC104428449			31565	-3.474	-0.0964	Yes
278	120286925	LOC120286925			31584	-3.524	-0.0865	Yes
279	104427367	LOC104427367			31592	-3.539	-0.0763	Yes
280	120291996	LOC120291996			31724	-3.959	-0.0687	Yes
281	104414044	LOC104414044			31725	-3.961	-0.057	Yes
282	104440094	LOC104440094			31774	-4.158	-0.0462	Yes
283	120289670	LOC120289670			31779	-4.173	-0.0339	Yes
284	104427055	LOC104427055			31787	-4.214	-0.0217	Yes
285	104420736	LOC104420736			31794	-4.251	-0.0093	Yes
286	104419546	LOC104419546			31932	-4.925	0.0009	Yes

Table S5. Genes in terpene synthase activity (GO-GSEA).

	PROBE	DESCRIPTION (from dataset)	GENE SYMBOL	GENE_TITLE	RANK IN GENE LIST	RANK METRIC SCORE	RUNNING ES	CORE ENRICHMENT
1	104435491	LOC104435491			3	4.995	0.0212	Yes
2	120292023	LOC120292023			9	4.988	0.0424	Yes
3	104431307	LOC104431307			17	4.981	0.0635	Yes
4	104442026	LOC104442026			21	4.98	0.0846	Yes
5	104426794	LOC104426794			44	4.965	0.1052	Yes
6	120289312	LOC120289312			60	4.954	0.1259	Yes
7	104450191	LOC104450191			68	4.948	0.1468	Yes
8	104422073	LOC104422073			77	4.944	0.1677	Yes
9	104423654	LOC104423654			103	4.93	0.1879	Yes
10	108958990	LOC108958990			207	4.83	0.2053	Yes
11	120290740	LOC120290740			323	4.667	0.2216	Yes
12	104442836	LOC104442836			472	4.408	0.2358	Yes
13	104442834	LOC104442834			538	4.308	0.2522	Yes

14	104431518	LOC104431518			648	4.148	0.2664	Yes
15	104454904	LOC104454904			701	4.045	0.2821	Yes
16	104452558	LOC104452558			743	3.973	0.2978	Yes
17	104456309	LOC104456309			747	3.963	0.3146	Yes
18	104429381	LOC104429381			792	3.884	0.3298	Yes
19	104452559	LOC104452559			808	3.867	0.3459	Yes
20	104452556	LOC104452556			839	3.804	0.3612	Yes
21	104435858	LOC104435858			866	3.757	0.3764	Yes
22	104454860	LOC104454860			921	3.688	0.3905	Yes
23	104435401	LOC104435401			953	3.649	0.4051	Yes
24	104454882	LOC104454882			954	3.649	0.4207	Yes
25	120288878	LOC120288878			1147	3.367	0.429	Yes
26	120289974	LOC120289974			1168	3.344	0.4427	Yes
27	104435460	LOC104435460			1374	3.135	0.4496	Yes
28	120292774	LOC120292774			1399	3.112	0.4621	Yes
29	104427170	LOC104427170			2012	2.53	0.4536	Yes
30	120289357	LOC120289357			2187	2.418	0.4585	Yes
31	104435451	LOC104435451			2191	2.418	0.4687	Yes

Table S6. Genes in carbon-oxygen lyase activity, acting on phosphates (GO-GSEA).

	PROBE	DESCRIPTION (from dataset)	GENE SYMBOL	GENE_TITLE	RANK IN GENE LIST	RANK METRIC SCORE	RUNNING ES	CORE ENRICHMENT
1	104435491	LOC104435491			3	4.995	0.021	Yes
2	120292023	LOC120292023			9	4.988	0.0419	Yes
3	104431307	LOC104431307			17	4.981	0.0627	Yes
4	104442026	LOC104442026			21	4.98	0.0836	Yes
5	104426794	LOC104426794			44	4.965	0.1039	Yes
6	120289312	LOC120289312			60	4.954	0.1243	Yes
7	104450191	LOC104450191			68	4.948	0.145	Yes
8	104422073	LOC104422073			77	4.944	0.1656	Yes
9	104423654	LOC104423654			103	4.93	0.1856	Yes
10	108958990	LOC108958990			207	4.83	0.2028	Yes
11	120290740	LOC120290740			323	4.667	0.2189	Yes
12	104442836	LOC104442836			472	4.408	0.2328	Yes
13	104442834	LOC104442834			538	4.308	0.2489	Yes
14	104431518	LOC104431518			648	4.148	0.263	Yes
15	104454904	LOC104454904			701	4.045	0.2784	Yes
16	104452558	LOC104452558			743	3.973	0.2939	Yes
17	104456309	LOC104456309			747	3.963	0.3106	Yes
18	104429381	LOC104429381			792	3.884	0.3256	Yes
19	104452559	LOC104452559			808	3.867	0.3414	Yes
20	104452556	LOC104452556			839	3.804	0.3565	Yes

21	104435858	LOC104435858			866	3.757	0.3716	Yes
22	104454860	LOC104454860			921	3.688	0.3854	Yes
23	104435401	LOC104435401			953	3.649	0.3999	Yes
24	104454882	LOC104454882			954	3.649	0.4153	Yes
25	120288878	LOC120288878			1147	3.367	0.4234	Yes
26	120289974	LOC120289974			1168	3.344	0.4369	Yes
27	104435460	LOC104435460			1374	3.135	0.4437	Yes
28	120292774	LOC120292774			1399	3.112	0.4561	Yes
29	104427170	LOC104427170			2012	2.53	0.4475	Yes
30	120289357	LOC120289357			2187	2.418	0.4522	Yes
31	104435451	LOC104435451			2191	2.418	0.4623	Yes

Table S7. Genes in carbon-oxygen lyase activity (GO-GSEA).

	PROBE	DESCRIPTION (from dataset)	GENE SYMBOL	GENE_TITLE	RANK IN GENE LIST	RANK METRIC SCORE	RUNNING ES	CORE ENRICHMENT
1	104435491	LOC104435491			3	4.995	0.0188	Yes
2	120292023	LOC120292023			9	4.988	0.0375	Yes
3	104431307	LOC104431307			17	4.981	0.0561	Yes
4	104442026	LOC104442026			21	4.98	0.0748	Yes
5	104426794	LOC104426794			44	4.965	0.0929	Yes
6	120289312	LOC120289312			60	4.954	0.1112	Yes
7	104450191	LOC104450191			68	4.948	0.1297	Yes
8	104422073	LOC104422073			77	4.944	0.1481	Yes
9	104423654	LOC104423654			103	4.93	0.166	Yes
10	108958990	LOC108958990			207	4.83	0.181	Yes
11	120290740	LOC120290740			323	4.667	0.195	Yes
12	104442836	LOC104442836			472	4.408	0.207	Yes
13	104442834	LOC104442834			538	4.308	0.2212	Yes
14	104431518	LOC104431518			648	4.148	0.2335	Yes
15	104454904	LOC104454904			701	4.045	0.2472	Yes
16	104452558	LOC104452558			743	3.973	0.2609	Yes
17	104456309	LOC104456309			747	3.963	0.2758	Yes
18	104429381	LOC104429381			792	3.884	0.2891	Yes
19	104452559	LOC104452559			808	3.867	0.3032	Yes
20	104452556	LOC104452556			839	3.804	0.3167	Yes
21	104435858	LOC104435858			866	3.757	0.33	Yes
22	104454860	LOC104454860			921	3.688	0.3423	Yes
23	104435401	LOC104435401			953	3.649	0.3551	Yes
24	104454882	LOC104454882			954	3.649	0.3689	Yes
25	104430995	LOC104430995			1018	3.556	0.3804	Yes
26	120288878	LOC120288878			1147	3.367	0.3891	Yes
27	120289974	LOC120289974			1168	3.344	0.4011	Yes

28	104435460	LOC104435460			1374	3.135	0.4065	Yes
29	120292774	LOC120292774			1399	3.112	0.4175	Yes
30	104427170	LOC104427170			2012	2.53	0.4077	Yes
31	120289357	LOC120289357			2187	2.418	0.4114	Yes
32	104435451	LOC104435451			2191	2.418	0.4204	Yes
33	104421260	LOC104421260			2578	2.22	0.4167	Yes
34	104427202	LOC104427202			2627	2.199	0.4235	Yes
35	104423547	LOC104423547			3018	1.985	0.4187	Yes
36	104422552	LOC104422552			3120	1.935	0.4228	Yes
37	104438521	LOC104438521			3481	1.777	0.4181	Yes
38	104445701	LOC104445701			3549	1.746	0.4226	Yes
39	104456834	LOC104456834			3561	1.742	0.4289	Yes

Table S8. Genes in sesquiterpenoid and triterpenoid biosynthesis (KEGG-GSEA).

	PROBE	DESCRIPTION (from dataset)	GENE SYMBOL	GENE_TITLE	RANK IN GENE LIST	RANK METRIC SCORE	RUNNING ES	CORE ENRICHMENT
1	novel.111	-			97	4.682	0.057	Yes
2	108958990	LOC108958990			135	4.472	0.1133	Yes
3	104431518	LOC104431518			250	3.819	0.1587	Yes
4	104423654	LOC104423654			273	3.708	0.2056	Yes
5	104442834	LOC104442834			310	3.49	0.2493	Yes
6	104442836	LOC104442836			418	3.106	0.2858	Yes
7	104438521	LOC104438521			488	2.911	0.321	Yes
8	104450191	LOC104450191			716	2.45	0.3453	Yes
9	120289357	LOC120289357			718	2.446	0.3767	Yes
10	104438524	LOC104438524			748	2.411	0.4067	Yes
11	104431307	LOC104431307			752	2.403	0.4374	Yes
12	104422073	LOC104422073			877	2.239	0.4623	Yes
13	104450716	LOC104450716			951	2.152	0.4876	Yes
14	120289312	LOC120289312			1114	1.999	0.5082	Yes
15	104447805	LOC104447805			1427	1.763	0.521	Yes
16	104445174	LOC104445174			1595	1.665	0.5372	Yes
17	104418420	LOC104418420			1763	1.561	0.552	Yes
18	104454355	LOC104454355			1920	1.494	0.5662	Yes
19	104426794	LOC104426794			2300	1.335	0.5715	Yes
20	120289974	LOC120289974			2304	1.335	0.5885	Yes
21	104418125	LOC104418125			2442	1.288	0.6008	Yes
22	104449232	LOC104449232			2756	1.184	0.6062	Yes
23	104447373	LOC104447373			3071	1.097	0.6104	Yes

Table S9. Genes in phenylpropanoid biosynthesis (KEGG-GSEA).

	PROBE	DESCRIPTION (from dataset)	GENE SYMBOL	GENE_TITLE	RANK IN GENE LIST	RANK METRIC SCORE	RUNNING ES	CORE ENRICHMENT
1	104445601	LOC104445601			4	4.999	0.0094	Yes
2	104445600	LOC104445600			16	4.997	0.0186	Yes
3	104453856	LOC104453856			31	4.996	0.0276	Yes
4	104438294	LOC104438294			88	4.986	0.0354	Yes
5	104426975	LOC104426975			146	4.976	0.0431	Yes
6	104456851	LOC104456851			243	4.954	0.0495	Yes
7	104453855	LOC104453855			349	4.93	0.0556	Yes
8	104452254	LOC104452254			395	4.914	0.0636	Yes
9	104415687	LOC104415687			400	4.913	0.0728	Yes
10	104415688	LOC104415688			405	4.91	0.082	Yes
11	104454445	LOC104454445			414	4.908	0.0911	Yes
12	104455395	LOC104455395			415	4.908	0.1005	Yes
13	104449485	LOC104449485			460	4.889	0.1084	Yes
14	104444152	LOC104444152			464	4.887	0.1176	Yes
15	104453315	LOC104453315			551	4.848	0.1242	Yes
16	104443712	LOC104443712			555	4.847	0.1333	Yes
17	104455132	LOC104455132			654	4.811	0.1394	Yes
18	104455140	LOC104455140			655	4.81	0.1486	Yes
19	104422587	LOC104422587			886	4.709	0.1504	Yes
20	104430095	LOC104430095			887	4.708	0.1593	Yes
21	104422800	LOC104422800			1016	4.645	0.1642	Yes
22	104433339	LOC104433339			1017	4.645	0.173	Yes
23	104418455	LOC104418455			1133	4.587	0.1782	Yes
24	104437652	LOC104437652			1156	4.574	0.1862	Yes
25	104437651	LOC104437651			1220	4.532	0.1929	Yes
26	104433684	LOC104433684			1272	4.507	0.1999	Yes
27	104454440	LOC104454440			1450	4.398	0.2027	Yes
28	104450635	LOC104450635			1506	4.359	0.2093	Yes
29	104456852	LOC104456852			1532	4.341	0.2168	Yes
30	104441575	LOC104441575			1832	4.185	0.2155	Yes
31	120291593	LOC120291593			1970	4.094	0.219	Yes
32	104437195	LOC104437195			1974	4.089	0.2267	Yes
33	104450510	LOC104450510			1992	4.08	0.2339	Yes
34	104431840	LOC104431840			2145	3.986	0.2368	Yes
35	104438293	LOC104438293			2173	3.969	0.2435	Yes
36	120294767	LOC120294767			2254	3.92	0.2484	Yes
37	120296049	LOC120296049			2264	3.913	0.2556	Yes
38	104445602	LOC104445602			2272	3.906	0.2628	Yes
39	104437647	LOC104437647			2332	3.868	0.2684	Yes
40	120296044	LOC120296044			2429	3.812	0.2726	Yes
41	104417080	LOC104417080			2538	3.744	0.2764	Yes
42	104450451	LOC104450451			2553	3.736	0.2831	Yes
43	104430395	LOC104430395			2563	3.733	0.2899	Yes

44	104455389	LOC104455389			2636	3.688	0.2947	Yes
45	104441401	LOC104441401			2685	3.655	0.3001	Yes
46	104416462	LOC104416462			2690	3.651	0.307	Yes
47	104443731	LOC104443731			2770	3.601	0.3114	Yes
48	120294448	LOC120294448			2829	3.564	0.3163	Yes
49	104431976	LOC104431976			2833	3.563	0.323	Yes
50	120296047	LOC120296047			3010	3.469	0.3241	Yes
51	104419698	LOC104419698			3158	3.389	0.326	Yes
52	104455394	LOC104455394			3198	3.368	0.3312	Yes
53	104417991	LOC104417991			3213	3.361	0.3372	Yes
54	104456751	LOC104456751			3231	3.352	0.343	Yes
55	104453243	LOC104453243			3268	3.334	0.3483	Yes
56	104442380	LOC104442380			3298	3.324	0.3537	Yes
57	120294132	LOC120294132			3311	3.316	0.3596	Yes
58	120296045	LOC120296045			3352	3.296	0.3646	Yes
59	120296048	LOC120296048			3518	3.21	0.3656	Yes
60	104437427	LOC104437427			3594	3.169	0.3693	Yes

Table S10. Genes in plant-pathogen interaction (KEGG-GSEA).

	PROBE	DESCRIPTION (from dataset)	GENE SYMBOL	GENE_TITLE	RANK IN GENE LIST	RANK METRIC SCORE	RUNNING ES	CORE ENRICHMENT
177	104449494	LOC104449494			27613	-1.939	-0.4521	Yes
178	104416970	LOC104416970			27628	-1.945	-0.4491	Yes
179	104422137	LOC104422137			27672	-1.962	-0.4471	Yes
180	104414302	LOC104414302			27711	-1.975	-0.4448	Yes
181	120287568	LOC120287568			27727	-1.98	-0.4418	Yes
182	104414483	LOC104414483			27758	-1.99	-0.4393	Yes
183	104421232	LOC104421232			27869	-2.029	-0.4392	Yes
184	104443414	LOC104443414			27960	-2.061	-0.4384	Yes
185	104444272	LOC104444272			28021	-2.089	-0.4366	Yes
186	104433916	LOC104433916			28067	-2.105	-0.4344	Yes
187	104456380	LOC104456380			28105	-2.117	-0.4318	Yes
188	104448458	LOC104448458			28180	-2.146	-0.4304	Yes
189	104423255	LOC104423255			28299	-2.195	-0.4303	Yes
190	104443940	LOC104443940			28364	-2.22	-0.4284	Yes
191	104448722	LOC104448722			28415	-2.246	-0.426	Yes
192	104443936	LOC104443936			28429	-2.252	-0.4225	Yes
193	104419928	LOC104419928			28507	-2.288	-0.4209	Yes
194	104423668	LOC104423668			28511	-2.289	-0.417	Yes
195	104433709	LOC104433709			28536	-2.297	-0.4138	Yes
196	104445156	LOC104445156			28599	-2.331	-0.4117	Yes
197	120285870	LOC120285870			28608	-2.335	-0.4078	Yes
198	104456273	LOC104456273			28656	-2.357	-0.4052	Yes

199	104444452	LOC104444452	28663	-2.361	-0.4013	Yes
200	120286843	LOC120286843	28668	-2.362	-0.3973	Yes
201	104441710	LOC104441710	28766	-2.41	-0.3961	Yes
202	104428053	LOC104428053	28767	-2.411	-0.3919	Yes
203	104456403	LOC104456403	28836	-2.442	-0.3898	Yes
204	104419657	LOC104419657	28966	-2.51	-0.3894	Yes
205	104451514	LOC104451514	28984	-2.522	-0.3856	Yes
206	104421651	LOC104421651	29059	-2.559	-0.3834	Yes
207	104426329	LOC104426329	29167	-2.624	-0.3822	Yes
208	104422924	LOC104422924	29193	-2.64	-0.3784	Yes
209	104448798	LOC104448798	29200	-2.644	-0.3739	Yes
210	104421646	LOC104421646	29251	-2.668	-0.3709	Yes
211	120293261	LOC120293261	29314	-2.696	-0.3681	Yes
212	104441713	LOC104441713	29395	-2.738	-0.3658	Yes
213	104438492	LOC104438492	29460	-2.769	-0.363	Yes
214	104444268	LOC104444268	29477	-2.778	-0.3586	Yes
215	108960305	LOC108960305	29491	-2.785	-0.3542	Yes
216	120287703	LOC120287703	29610	-2.86	-0.3529	Yes
217	104450406	LOC104450406	29634	-2.873	-0.3486	Yes
218	104432889	LOC104432889	29653	-2.88	-0.3442	Yes
219	104419049	LOC104419049	29656	-2.881	-0.3392	Yes
220	104450465	LOC104450465	29718	-2.924	-0.336	Yes
221	104443368	LOC104443368	29739	-2.937	-0.3315	Yes
222	104429436	LOC104429436	29742	-2.938	-0.3265	Yes
223	104437953	LOC104437953	29836	-2.997	-0.3241	Yes
224	104427319	LOC104427319	29870	-3.02	-0.3199	Yes
225	120287698	LOC120287698	29978	-3.1	-0.3178	Yes
226	104425716	LOC104425716	29980	-3.101	-0.3125	Yes
227	104443369	LOC104443369	30053	-3.141	-0.3092	Yes
228	104456341	LOC104456341	30127	-3.206	-0.3059	Yes
229	104425342	LOC104425342	30159	-3.225	-0.3013	Yes
230	104456407	LOC104456407	30207	-3.257	-0.2971	Yes
231	104454049	LOC104454049	30269	-3.294	-0.2932	Yes
232	104437523	LOC104437523	30271	-3.295	-0.2875	Yes
233	120286810	LOC120286810	30337	-3.339	-0.2837	Yes
234	104426306	LOC104426306	30371	-3.378	-0.2789	Yes
235	104442385	LOC104442385	30430	-3.425	-0.2747	Yes
236	104444270	LOC104444270	30451	-3.442	-0.2693	Yes
237	104425417	LOC104425417	30471	-3.46	-0.2639	Yes
238	104449707	LOC104449707	30528	-3.507	-0.2596	Yes
239	120287409	LOC120287409	30545	-3.519	-0.2539	Yes
240	104416990	LOC104416990	30572	-3.536	-0.2486	Yes
241	104442040	LOC104442040	30659	-3.606	-0.245	Yes
242	120295517	LOC120295517	30666	-3.611	-0.2389	Yes
243	104422003	LOC104422003	30690	-3.636	-0.2333	Yes
244	104453847	LOC104453847	30833	-3.754	-0.2311	Yes
245	104448267	LOC104448267	30852	-3.77	-0.2251	Yes
246	104425834	LOC104425834	30859	-3.775	-0.2187	Yes
247	104417001	LOC104417001	31014	-3.91	-0.2167	Yes

248	104422757	LOC104422757			31034	-3.927	-0.2105	Yes
249	120294306	LOC120294306			31045	-3.937	-0.2039	Yes
250	104423509	LOC104423509			31081	-3.976	-0.1981	Yes
251	120293645	LOC120293645			31096	-3.988	-0.1916	Yes
252	104456406	LOC104456406			31187	-4.061	-0.1873	Yes
253	104444445	LOC104444445			31198	-4.07	-0.1805	Yes
254	104446729	LOC104446729			31254	-4.118	-0.1751	Yes
255	104442133	LOC104442133			31304	-4.163	-0.1694	Yes
256	104427433	LOC104427433			31328	-4.185	-0.1628	Yes
257	120294405	LOC120294405			31339	-4.197	-0.1558	Yes
258	104451769	LOC104451769			31426	-4.276	-0.151	Yes
259	104429873	LOC104429873			31465	-4.309	-0.1447	Yes
260	104444068	LOC104444068			31472	-4.313	-0.1374	Yes
261	104425137	LOC104425137			31510	-4.354	-0.1309	Yes
262	104444418	LOC104444418			31521	-4.365	-0.1237	Yes
263	104422220	LOC104422220			31535	-4.372	-0.1164	Yes
264	104428600	LOC104428600			31541	-4.383	-0.109	Yes
265	104443366	LOC104443366			31568	-4.412	-0.1021	Yes
266	104454739	LOC104454739			31570	-4.413	-0.0944	Yes
267	104425347	LOC104425347			31580	-4.416	-0.087	Yes
268	104444265	LOC104444265			31586	-4.426	-0.0795	Yes
269	120294824	LOC120294824			31623	-4.458	-0.0728	Yes
270	104453031	LOC104453031			31629	-4.461	-0.0652	Yes
271	104450370	LOC104450370			31637	-4.464	-0.0577	Yes
272	104426344	LOC104426344			31639	-4.466	-0.0499	Yes
273	120293654	LOC120293654			31643	-4.47	-0.0422	Yes
274	104448346	LOC104448346			31644	-4.47	-0.0344	Yes
275	104434125	LOC104434125			31660	-4.481	-0.0271	Yes
276	104445200	LOC104445200			31701	-4.51	-0.0205	Yes
277	104440808	LOC104440808			31704	-4.513	-0.0127	Yes
278	104426307	LOC104426307			31756	-4.559	-0.0063	Yes
279	104444267	LOC104444267			32042	-4.796	-0.0069	Yes
280	104456528	LOC104456528			32073	-4.816	0.0006	Yes
281	104444424	LOC104444424			32100	-4.832	0.0082	Yes

Table S11. Genes in flavonoid biosynthesis (KEGG-GSEA).

	PROBE	DESCRIPTION (from dataset)	GENE SYMBOL	GENE_TITLE	RANK IN GENE LIST	RANK METRIC SCORE	RUNNING ES	CORE ENRICHMENT
1	104456052	LOC104456052			13	4.998	0.0227	Yes
2	104426975	LOC104426975			146	4.976	0.0416	Yes
3	104415687	LOC104415687			400	4.913	0.0565	Yes
4	104415688	LOC104415688			405	4.91	0.079	Yes

5	104455395	LOC104455395	415	4.908	0.1014	Yes
6	104415137	LOC104415137	470	4.886	0.1223	Yes
7	104443712	LOC104443712	555	4.847	0.1421	Yes
8	104455132	LOC104455132	654	4.811	0.1613	Yes
9	104455140	LOC104455140	655	4.81	0.1836	Yes
10	104428803	LOC104428803	666	4.806	0.2055	Yes
11	120294781	LOC120294781	692	4.794	0.2268	Yes
12	104430095	LOC104430095	887	4.708	0.2426	Yes
13	120294785	LOC120294785	1052	4.624	0.2589	Yes
14	104432776	LOC104432776	1380	4.441	0.2693	Yes
15	104450635	LOC104450635	1506	4.359	0.2855	Yes
16	104449845	LOC104449845	1884	4.149	0.293	Yes
17	104431840	LOC104431840	2145	3.986	0.3034	Yes
18	120294767	LOC120294767	2254	3.92	0.3182	Yes
19	120296049	LOC120296049	2264	3.913	0.336	Yes
20	120296044	LOC120296044	2429	3.812	0.3485	Yes
21	104455389	LOC104455389	2636	3.688	0.3592	Yes
22	120290178	LOC120290178	2737	3.626	0.3728	Yes
23	104443731	LOC104443731	2770	3.601	0.3885	Yes
24	120294448	LOC120294448	2829	3.564	0.4031	Yes
25	104431976	LOC104431976	2833	3.563	0.4195	Yes
26	120296047	LOC120296047	3010	3.469	0.4301	Yes
27	104455394	LOC104455394	3198	3.368	0.4399	Yes
28	104453243	LOC104453243	3268	3.334	0.4531	Yes
29	120296045	LOC120296045	3352	3.296	0.4658	Yes
30	120296048	LOC120296048	3518	3.21	0.4755	Yes
31	104420450	LOC104420450	3735	3.085	0.4831	Yes
32	104432774	LOC104432774	3829	3.031	0.4942	Yes
33	104454463	LOC104454463	3974	2.951	0.5034	Yes
34	104436514	LOC104436514	4301	2.805	0.5062	Yes
35	104441153	LOC104441153	4394	2.761	0.5161	Yes
36	104441822	LOC104441822	4981	2.521	0.5096	Yes
37	104450636	LOC104450636	4985	2.52	0.5212	Yes

Table S12. WGCNA disease resistance related module genes.

EC338 positive correlation		EC333 negative correlation	EC338 negative correlation with EC333 positive correlation
MEpink	MEtan	MEgreenyellow	MEsaddlebrown
120291506	104453526	104436533	104448928
104449885	104453361	104430936	104445252
104450993	104424635	104456799	104424752
104437752	104441683	104425869	104426217
104426863	104438376	104422794	104419057

104414247	104420006	104438573	104442219
104456301	104436363	104432842	104450478
104448798	104416141	104448851	104418785
104422496	104421812	104454201	120285888
104453055	104424700	104433910	104423290
104436548	104453561	104424743	104421540
104432557	104450852	104418884	104426756
120286881	104426822	104456398	104440718
104438399	104424758	104425364	104442362
104422285	104433348	104422657	104426238
104426754	104432639	104453437	104415307
104457395	104422776	104454528	104448299
104449214	104424352	104422772	104436313
104436828	104424857	104414528	104437500
104437634	104418942	104450253	104419579
104432680	104448773	104449883	104444319
104449932	104446877	104448922	104443488
104433742	104415854	104453387	104442156
104445600	104426272	104421700	104422238
104438051	104438200	104454478	104418194
120286245	104418682	104422033	104433932
104448025	104445410	104415747	104422733
104453382	104424281	104434133	104449414
104433374	104425564	104449524	104449514
104427965	104415970	104449017	104434231
104437235	104449269	104441483	104443645
104424418	104454685	104453137	104419491
104437563	104449552	104415636	104445334
104442991	104424607	104418419	104449108
104450773	104450696	104421278	104414803
104415766	104424265	104418447	104437444
104426029	104436026	104423234	104437686
104418982	104427640	104419004	104452645
104423225	104438362	104442024	104439186
104454440	104426686	104423010	104418438
104437963	104448228	104421366	104418123
104433743	104438032	104445221	104421174
104449880	104436102	104449346	104448238
104443648	104443998	104457394	104453159
104445011	120295714	104443370	104445581
120290849	120290287	104444274	104434797
104443766	104447964	104448687	104415261
104436144	104419394	104449800	104437139
104441236	104432674	104456509	104424280
104436438	104453664	104450348	104454643
104424824	104419153	108957826	104422167
104453344	104434227	104422205	104423517
104448513	108953833	104448621	104433230

104441814	104453006	104449037	104415355
104421865	104438956	104457257	104440607
104441984	104432695	104440995	104438177
104450695	104442338	104415966	104432922
104418469	120286445	104457306	104421365
104421518	104419452	104436914	104425409
104440778	104441822	104454242	104453569
104437522	104449364	104437360	104454575
104422374	104449641	104435840	novel.947
104456998	104425622	104448334	104426834
104419496	104418972	104441438	104415216
104415921	104433644	120288118	104419946
104421465	104444640	104456107	104421694
104452661	104456901	104442291	104437756
104437431	104450778	104427041	104423200
104442372	104441782	104442314	104438995
104456057	104456818	104414087	104436631
104456708	104445331	104441203	104423281
104419878	104417657	104425375	104438076
104416031	104457369	104449867	104447678
104444123	120294343	104423630	104448964
104419151	104449594	104415777	104445486
104441735	104433170	104438025	120292498
104449254	104448131	novel.1622	104437412
104449581	104432980	104426582	104450743
104435040	104426089	104426164	104438321
104423243	104419274	104422669	104454500
120295464	104449114	104437575	104442188
104438128	104441118	104443465	104454956
104453990	108960239	104430240	104428484
104425616	104428535	104421324	104424701
104426317	104456553	104415502	104439201
104420028	104424445	104424517	104449028
104457015	104423111	104423871	120290955
104415031	104448335	104435888	104418598
104441401	104454319	104453630	104452636
104419488	104454512	novel.1035	104438235
104456282	104424755	104423177	104440862
104444805	104450385	104442162	104421241
104423801	104434095	104450226	104451300
104421292	104453654	novel.1162	120289452
104434014	104418676	104449593	104422226
104421714	104450064	104425022	108953866
120293510	104432967	104443245	104437180
104445843	104454410	104419880	120290834
104418455	104456827	104418792	104438221
104433138	104418891	104432593	104429512
104446012	104422779	104430414	novel.1786

104421139	104437483	104433280	120291533
104414945	104422748	104447794	104423643
104418784	104424261	104436258	104448013
104452358	104445845	104422143	104431467
104433896	104423740	104422663	120294741
104453121	120289195	104440765	120288281
104435955	104421200	104419852	104453150
104436280	104450312	104427368	104438806
104450574	104425468	104419730	120286682
104426648	104424697	104437570	120286768
104456147	104424302	104452616	104440226
104414388	104425161	104448877	104435465
104447095	104436716	104442335	120285974
104457115	104425812	104445761	104418293
104449282	104450013	104414127	120288294
104444321	120294605	120288142	104448186
104419737	104447940	104448999	120286090
104415254	104442278	104437729	104448563
104421746	104414046	104433726	104430559
104452752	120291160	104418686	104420352
104421686	104433943	104448843	104435774
104426722	104450575	104440781	120292821
120293021	120290069	104426283	120287031
104433137	104441686	104420001	104425783
104443836	120294897	104426734	104422065
104422221	104438570	104415033	novel.830
104432738	104449715	104453319	104427367
104424918	104428397	104437851	104438851
104421802	104425497	104445681	104414763
104418708	104416611	104444620	104448296
104421137	104433973	120296185	104451036
104447907	104434075	104456227	104436956
104424333	104422792	120292416	104423665
104416327	104453329	104429023	104430446
104454204	104452637	104416868	104418513
104456240	104456492	104437681	104451390
104433769	104433901	104435574	104426577
104446106	104438391	104418440	104453212
104448985	120293168	104425856	104438120
104434287	104426632	104423265	104428921
104441375	104426293	120288698	120294229
120292245	104436521	104447772	104456234
104456122	104456484	104414421	104438296
104436767	104443475	120287260	104456391
104425112	104422269	104427023	120287661
120295413	104449680	104447349	104437528
120289247	120295346	120289506	104432772
104448338	120288078	120292883	104422002

104442305	104441030	104415926	120293320
104442344	104448969	104429094	108953880
104442397	108958510	104449919	104419938
104414680	104418296	104441630	104436516
104441617	104442134	104421770	120286602
104449461	104439941	104449949	120291535
104433041	104437276	108960176	novel.444
104444192	104432133	120292902	104418619
104454497	104457029	104450178	104415633
104453881	108958715	104429442	104437264
104453532	104423098	104418030	novel.186
104439635	104424578	104438227	104449034
104443984	104418132	104420259	104425171
104414235	104425738	104444829	120288673
104436298	104449735	104453135	120294662
104415167	120288985	120291256	108960346
104444209	104420566	108955632	104445860
104426849	104418242	novel.321	108953862
104445889	104456682	104448348	120291653
104442954	104437778	104425045	120289405
104450012	novel.78	novel.1216	104436777
104421048	104424796	104438632	104414132
104426678	104421655	104415589	104419000
104415826	104428685	novel.1913	104426568
104448128	104441857	104449078	104445518
104433914	108957790	120289205	novel.509
104441601	104427701	120287678	104423232
104436607	104425004	120294601	104447534
104441382	104422203	104441829	104438099
104415887	104453874	120288053	104447493
104448630	104429779	104445387	120291903
104449937	120289383	104456181	104448129
104425342	novel.1028	104441042	104452638
104438521	120295332	104451733	120286762
104433925	novel.1049	104418642	108954038
104425402	104419179	120290962	120286469
104418877	120289904	104422466	104436557
104454675	104422200	104456674	104451972
104449947	104436944	104443473	120289396
104448849	104432523	104421124	104441369
104433602	104418650	novel.487	104440380
120285843	120291194	104448345	120289413
104436370	104423148	120294126	104455371
104435993	104425580	104426611	104434037
104454487	120293249	104429607	104423808
104443460	novel.1524	104441501	104443861
104426019	104451346	104425702	104435980
104443714	104452353	104436735	104416167

120292225	120286157	104436493	104444677
104445742	104417796	104428899	104422120
104450693	108953817	104418377	104450674
104447214	104438578	104453837	104447063
104444095	104416959	104450513	120292471
104441673	104441379	104428618	120290268
104443724	108956857	104417826	
104441250	104414781	104426338	
104426336	120288966	104450047	
104415878	120288914	104414269	
104422236	120295478	104441942	
104421736	104445185	104424144	
104434077	104417002	104421989	
104421722	120288220	novel.1672	
104435770	120289124	104438329	
104415740	104433538	104424625	
104450034	120294014	104441202	
120286014	104454178	120291396	
104450509	104445857	104449665	
104450667	novel.1761	104436867	
104415531	104428611	104432193	
104419485	120286242	120295748	
104426286	104419584	9829715	
104452930	104441497	120292926	
104421829	104423055	108958880	
104425651	120287655	108959091	
104453024	104454103	104443967	
104433607	108955670	104417187	
104443684	108959095	104436198	
104452428	104442763	104441101	
104419602	120294301	104423023	
104452456	120289624	104421669	
104449958	104449203	104416625	
104440726	120286013	104448288	
104454001	104430513	104438524	
104436406	108955011	104449546	
104421249	104421565	108954914	
104445265	104451624	104440282	
104436736	120292169	novel.1194	
120287620	104456193	novel.447	
104420031	104456104	104434108	
104421790	120286873	104453923	
104444993	104426130	120288940	
104418308	104418387	104437754	
104436754	120292242	novel.814	
104421102	novel.178	104424619	
104422788	108955725	104441494	
104441065	108955753	104420904	

104441587	120296107	104431595
104424338	120288114	104437148
104436468	104420833	108955749
104438079	104422089	104457321
104453665	104448084	120292161
120294625	120291466	104414922
104438985	104414911	120296296
104426367	104436129	120291391
104414676	120291460	104430467
104441939	104437537	104420617
120289178	104426745	104437715
104421852	104415409	novel.1340
104425861	108954913	108961003
104449631	104457045	104436189
104436696	novel.1050	104440272
104456043	104423907	104444025
104452903	104443769	104436191
novel.1053	104422355	104424819
104441764	104436902	104421215
104447360	104418268	104438317
104420419	104440716	120291999
104431307	104440839	104438830
104444541	104432520	104449252
104424219	120290659	104437751
104432931	104442693	104414725
104425146	120291464	120287321
104421473	120287738	120292270
104421052	104424408	104418326
104448183	104422051	104426759
104456353	novel.393	novel.1603
104414105	novel.1521	104419706
104414444	104436133	104419346
104449420	novel.351	104449845
104423888	120292968	104419280
novel.348	104423735	104438794
120287701	120291470	120293988
104432545	novel.549	104449082
104445018	104448367	104452449
104435133	104456465	104436062
104418180	104454530	104418187
104444649	120291395	104452308
104421173	120287746	104438328
104445134	104418382	104419635
104420021	104443459	novel.245
104418841	novel.1436	120291511
104444305	104421653	104452254
104457043	novel.786	novel.341
104453907	108958989	novel.1812

104421572	104435778	novel.64
104432890	104449425	108954084
104454636	novel.1624	104422001
104441730	104451479	120287556
104434132	108958343	104436712
104444174	104415635	120296128
104438518	104445422	108957576
104441006	104452569	104456664
104419947	novel.1874	120286586
104443232	120291192	novel.1011
104438377	120291990	120286173
104422918	120294899	104431694
104454644	104452732	104425039
104444298	104451480	104438159
104440854	104424653	novel.1611
104426649	120293202	104441086
104433944	120293244	104428447
104452947	104418306	104437903
104454426	104444641	104434753
104441701	120291890	104438892
104421665	novel.790	104418223
104438388	104443095	104454467
104441908	novel.1712	120286221
104448493	novel.1056	104436809
104437443	104450555	104435768
104453930	120291734	104433582
104419527	104418564	novel.1868
104421466	104438113	104433105
120286196	104414823	104446525
104421464	104417498	104450638
104433218	104415571	104435951
104453520	120286824	novel.110
104442109	104421866	104431997
104421793	104431006	104436190
120293063	novel.1117	104423492
104422003	104437776	104425113
104443378	novel.838	104426173
104448785	120290470	120291336
104420000	120291458	104427525
104425739	104445275	novel.344
104433319	novel.209	108957973
104424312	120286195	novel.556
104416083	104439993	120292176
104426850	104421906	120293005
104445775	104453364	novel.478
120289189	108960054	104445636
104442332	120289508	104445616
104428381	104428756	novel.273

104414792	120289785	104456665
108957702	104440953	novel.1344
120287023	104425274	104436168
104437854	120288243	104436261
104453818	120293899	104414067
104424470	104448489	120289701
104434022	104451189	108959268
104448961	104441728	104418089
104457373	104431186	120286845
104441414	104418874	104428757
104414870	9829718	108956720
104434122	104421482	novel.1539
104415957	104429095	104430701
120289670	104426409	104453062
104433820	104456804	120287761
104422587	104445891	104421443
104441087	104451853	120294041
104415462	104426687	104456918
104433227	104441321	novel.304
104455844	104415770	104438421
104449120	120289855	104427055
104453097	104437840	novel.281
104415806	104451124	novel.1087
104416283	120286146	120289641
104454564	120285948	108956196
104429702	104448179	104449351
104436624	104426782	novel.1813
104426219	104434494	104417913
120292137	104436311	120290846
104425686	120289139	120291314
104445633	104415624	120294603
104436130	104422689	120286803
104434072	108961066	104436955
104436916	104449614	novel.1057
120286235	108958113	novel.813
104417570	108958328	120293513
104448008	104455363	104431916
104415733	120295539	104456964
104426650	108960908	120295263
104432917	104449124	108954808
104433660	120291472	novel.1602
104437863	104454080	104449330
104421170	104418450	108954120
104443716	104427134	120291497
104447761	104439456	104416376
104426098	104418687	104415403
120290966	104428868	120294815
104454031	120286879	104429379

104456931	120292972	104445488
104434789	104443888	104420034
104444812	104438962	104431986
104456021	104440039	108957177
104441398	120289802	120286418
104419905	108954479	104428528
120295973	120293487	104436885
104433722	104425763	104436884
104437847	108958664	104456081
104454591	120293253	120294600
104426860	120288894	120288421
104449938	120293530	novel.476
104441851	120291886	104451225
104433415	104443059	novel.514
104447927	104424107	novel.617
104445723	104454770	120292669
104442249	104447285	104422019
104416416	120289803	120287599
120290847	120289216	120289537
104456120	104452265	120292007
104440593	120296252	104418420
104453371		104445238
104441055		104427189
120294132		120292057
104418892		104436517
104453855		104417854
104447787		104429424
104414614		120295501
120296357		120291392
104444291		104453756
104448832		104449002
104424892		108957080
104418167		120294042
120291930		104439207
novel.1251		104435032
104427296		104429290
104454483		104428786
104449807		120292171
104425582		novel.1030
104441175		120293476
120286822		120289386
novel.1873		104440074
104442311		108954609
104423991		novel.276
104422358		104418080
104448987		104424639
104434165		104423024
104433617		104445567

104437552	104419743
104421523	104437517
104417099	104427772
104444295	104450542
104438395	104423757
104416101	108954769
104441850	104437314
104449689	104440509
104419776	104438728
104454461	120293318
104454202	120286900
104419262	104432945
104453400	104422442
104418508	104430502
104432937	120286785
104421049	104436527
104440830	104426075
104449911	104421345
104451665	120286056
104433308	9829636
104429929	38466617_part1
104415336	120294442
104443386	120291920
104421894	120287733
104450596	120289160
104418818	104431333
120292999	120294800
104438098	120294576
104422332	120292277
120293851	104423350
104418252	104448450
novel.1648	novel.627
104442104	104455686
108958990	104414503
104425053	104438281
104421219	104456359
104422384	104417714
104433556	104439790
108955120	104420552
104415282	104446404
104457358	120289414
104441608	104443921
104426853	104414119
104450615	104444931
104433359	104431491
104438369	104430593
104426588	104417852
104432991	104424855

104422095	104424674
104420192	104420137
120288334	120290347
104432908	120290399
104450191	104448357
104442066	104427993
104444419	104428233
104433292	104438156
104433562	108960378
108955706	120286482
104443957	104447144
104432778	120291063
104434064	104438944
104419444	104445925
104430707	104443885
104453723	104438622
104422462	104419478
108954252	104417360
104436071	104432919
104436546	
104445704	
104418156	
104455112	
104449357	
104452161	
104425031	
104440596	
novel.1142	
104433092	
120294896	
104423699	
104443606	
120294471	
104433826	
104424992	
104424656	
104445315	
104420857	
104425649	
104419006	
104438398	
104449842	
novel.1458	
novel.1504	
104419790	
104433614	
120289601	
104426308	

novel.398
novel.1838
104437042
104436212
104449285
104431454
104418236
104436431
104444115
104425227
104419464
104419078
104453381
104453971
104447991
104441271
104444480
104432614
104419698
104444487
104433812
104447797
104448458
104419325
104444096
104444877
104438305
104422531
novel.1700
104442065
120295957
104452195
104436314
novel.1207
120293269
104442080
104425033
104449380
novel.1136
104430313
104456483
104433729
104416113
104442026
104441418
104445807
104433165
104418273

104451555
104445511
104443601
120287019
104441787
120291657
104433493
104422530
104421948
104429238
104456902
104442198
104425845
104422659
104447703
104425302
104449319
104423019
104440321
104450618
novel.1137
104426419
104436462
104430323
120286617
104452381
104429125
104416596
104447573
120288332
108956654
104430495
104415892
novel.1337
104441910
104425340
104414487
104420479
120293526
104445719
104419789
104432803
104448702
104445739
120291994
104419892
104455546
120295972

120287110
104438776
120289527
104420963
104415608
120290965
104449232
104456324
104456709
104425698
108956893
104455997
120291369
104456498
novel.1250
104415947
104419005
104455563
104441058
104436730
104444806
104439484
novel.887
104424974
104439611
novel.685
104414549
104453401
104441493
120292109
120288313
104440727
104417766
104438512
104419600
104438645
104420141
104437406
104433525
104418894
104437753
104457402
104443531
104453841
104423594
104451317
104454024
104436644

120286991
104441653
104417741
novel.1178
104450251
104424629
104416285
104445586
104425392
104428135
104420204
104424580
104421912
104447805
novel.1529
104429228
104449541
120286005
104438236
120288728
104433766
novel.638
104449226
104428753
104444552
104441820
120290394
104424458
120290182
104436278
104441333
104447435
104435650
120286651
104414097
104429331
104434461
104443402
104414204
104445965
104450300
104417599
104450169
novel.670
108957819
104434035
120288071
104441444

104418389
104418680
120294630
120286511
novel.106
104416785
104430475
104438083
104453420
104448116
104431439
104445476
104423654
120292677
104435526
104433646
novel.1140
104445227
104430947
104447949
120291531
120294530
104437470
120294539
novel.1654
104416486
104416040
104455704
104424640
novel.1135
108954425
104455140
104443552
104418396
120295775
104445774
104443695
120291520
104455707
novel.300
104417605
104431276
104449217
104447808
104423921
104434117
120289877
104456134

104416744
120286807
104421335
104448042
104424197
104438367
104421728
104437023
104426066
104423367
104456819
104438297
104439225
104440524
104438756
104430774
104454065
104422367
120289712
104438081
104414159
104440429
104434791
104436407
novel.1302
104425563
104448765
104447931
104422787
104423595
104420496
104426771
104451109
104451166
novel.1289
novel.928
120292733
104445296
104441995
104447227
104436152
104449155
120290273
108960381
104419801
104456945
120289612
104442374

104443691
104414515
108956789
120292110
104429511
120291515
104457131
novel.1040
104431857
104438991
104421243
104445990
104456733
104454155
104418922
104429128
120292603
novel.1191
novel.1349
120291295
104425114
104423762
104435999
104442214
104423919
104450183
108958704
104437659
104430117
104452067
108955899
120288520
104445767
104449230
120288485
120287742
104440814
120295953
104453010
120289910
120286145
104420317
120295500
104424275
104449868
120291304
104435981
novel.929

104415923
120287279
104456839
104451104
120288733
104439017
104415475
104421454
104437348
104444083
120294727
104441318
104440824
120294778
104451138
104434242
120294786
120293508
104419674
120289001
104414772
104414181
104442609
104451900
104419648
104453692
104418251
104449701
104414612
120290950
104431262
120286396
104416876
120285959
104449771
108955043
104420794
120292437
120290878
120294485
120291388
104423723
104448833
104456218
104422535
104422255
108960232
120286739

104427948
104442707
104418481
104436848
104414179
104450570
120288411
120292561
120290822
120286132
104419569
104441643
120294540
104456571
104456802
104439562
104427046
120289671
104440427
120292033
104451448
104448633
104439949
120291807
104438885
120291450
104451204
104419784
104441963
104442848
104426829
120289357
104417751
104427840
104423565
120288900
120293748
104447689
104414165
104436826
104439610
104414634
104443466
104456212
104419360
104444484
104420710
104420948

104443244
120289417
104443203
120294784
104447732
104423373
104454651
104446587
104443197
120287472
120291967
120292675
120296308
120288867
104439723
104427801
104420863
104424111
104447621
104457262
104425021
104443292
104453424
104451734
104454998
